# Chemical Recycling of Silicones—Current State of Play (Building and Construction Focus)

**DOI:** 10.3390/polym16152220

**Published:** 2024-08-04

**Authors:** Andreas T. Wolf, Andreas Stammer

**Affiliations:** 1 Independent Researcher, 65510 Hünstetten, Germany; 2 Stammer Chemie GmbH, 55127 Mainz, Germany; andreas.stammer@stammer-chemie.com

**Keywords:** silicone, elastomer, chemical recycling, depolymerization

## Abstract

As the demand for silicone polymers continues to grow across various industries, the need for effective recycling methods has become increasingly important, because recycling silicone products reduces landfill waste, conserves resources, and uses less energy. Chemical recycling involves the depolymerization of silicone waste into oligomers, which can then be used to produce virgin-grade silicone. While this sector of the recycling industry is still in its infancy—we estimate that 35,000 to 45,000 metric tons of silicone waste will be chemically recycled worldwide in 2024—an increasing number of companies are beginning to explore the implementation of closed-loop systems to recycle silicones. This article examines the technical options and challenges for recycling silicone polymers, the major degradation chemistries available for depolymerizing silicones, and the current industrial reality of chemical recycling of silicones.

## 1. Introduction

Polysiloxanes, or silicones, are synthetic polymers with a silicon–oxygen backbone, as in silica (silicon dioxide), but with organic groups, typically methyl groups, attached to the silicon atoms. Depending on the molecular weight and structure of the polymer, silicone products are manufactured as liquids (fluids), gels, resins, elastomers (including adhesives and sealants), and emulsions. Silicone elastomers can be classified as either room temperature vulcanized (RTV) or high temperature vulcanized (HTV) silicones, depending on whether the crosslinking (curing) occurs at room temperature or higher temperatures. The majority of RTV products are cured with tri- and tetrafunctional silanes that form crosslinks, whereas HTV elastomers are generally cured with peroxides via a free radical mechanism. Liquid silicone rubbers (LSRs) are another class of elastomers. They are cured by a polyaddition (hydrosilylation) mechanism in which vinyl and hydride-substituted siloxane groups react to form an ethylene crosslink.

During the past eighty years, silicones have become ubiquitous materials due to their exceptional physical and chemical stability, their inertness, durability, and unique use properties as well as their low toxicity. Many of the unique properties of silicones can be attributed to the electronic nature of the siloxane bond, which is strongly influenced by the hyper-conjugative interactions between the free electron pair orbitals on the oxygen atoms and anti-bonding σ-orbitals of the Si-R bond, reducing the basicity of the oxygen atom and its reactivity toward electrophiles [1,2,3].

Silicones are widely used in a variety of end-use industries, including building and construction, electronics, transportation, healthcare, personal care, consumer goods, energy, and industrial processes. Most of these products are based on polydimethylsiloxane (PDMS). Also used as a base polymer, but to a much lesser extent, is polymethylphenyl-siloxane (PMPS).

Global annual production of silicones is growing at 5–6% per year and is expected to reach 2.9–3.0 million metric tons this year (2024), representing USD 20–22 billion in sales. The region with the highest sales is Asia, followed by Europe and North America. Elastomers have the highest share of sales in Asia (at around 70%), while oils/greases and elastomers have roughly equal shares of sales in Europe and North America [4,5].

While silicone is often a better, more durable material that generates less waste and greenhouse gases during its useful life than alternative materials (According to a study commissioned by the Global Silicone Council (GSC) (https://www.siliconescarbonbalance.eu/, accessed on 25 April 2024), the use of silicones (siloxanes and silanes) results in energy savings and greenhouse gas emission reductions that exceed the impact of manufacturing and end-of-life disposal by a factor of nine. The study found that silicone products in Europe, North America, and Japan reduce CO_2_ emissions by approximately 52 million tons annually), the continued growth of silicone waste underscores the need for an environmentally responsible way to dispose of, reuse, or recycle silicone products at the end of their useful life. Currently, silicone waste is either disposed of in landfills or incinerated. Since silicone is not biodegradable, the preferred option is to incinerate silicone (typically with household waste), which produces carbon dioxide, water, and amorphous silica. While amorphous silica poses far less of a health risk than crystalline silica [6], disposing of silicone products by incineration, also known as waste-to-energy conversion, still represents a loss of valuable resources, both in terms of the silicone material itself and the energy used to produce it.

To minimize waste and its impact on the environment, and to reduce dependence on virgin resources, companies are now beginning to explore the adoption of closed-loop systems based on the chemical recycling of silicone feedstocks. In this article, we will explore the technical options for depolymerizing silicones as well as the current industrial reality of chemical recycling of silicones on a global scale.

Compared to previous reviews by Rupasinghe [7,8], Rupasinghe and Furgal [9], Muzafarov et al. [10], and Elmanovich et al. [11], this review provides a more recent and comprehensive perspective on the chemical recycling of silicone products, with a particular focus on the industrial applicability of the proposed processes for silicone products sold to the construction industry, by critically evaluating the various claims regarding novel recycling routes.

Although previous research claims to be tied to industry needs to ensure real-world relevance, only few specific recycling methods have made it into industrial practice. In particular, the recycling of calcium carbonate-filled silicone sealants—the largest group of silicone materials used in the construction industry—has received little attention until very recently. As a result, previous review articles do not address the challenges associated with recycling these materials. Similarly, the review articles to date do not mention the methods and materials used in current commercial recycling practices.

There are two key reasons for the lack of large-scale, reliable industrial recycling processes: first, previous research on depolymerization chemistry has mainly used linear, non-crosslinked PDMS polymers as feedstock rather than a range of industrial products, and, thus, failed to identify critical process steps, and second, depolymerization chemistry has often been evaluated as a stand-alone process rather than its full integration into a closed-loop recycling system.

However, the visionary dream of chemical recycling and/or upcycling silicone polymers into value-added products has gained strong momentum in recent years. Partnership/alliance activities between start-ups and established companies have begun to emerge. Collaboration and co-development between these companies is increasing, a trend that we expect will lead to better integration of recycling into the value chains of large corporations in the future.

A substantial number of processes for depolymerizing silicone polymers have been developed. Future research interest must now focus on the ability to process multi-component industrial silicone materials in low-cost, closed-loop recycling processes that minimize energy requirements (balance of temperature versus reaction time), mass loss, and environment impact.

We hope that this paper will stimulate new ideas and open new avenues in the recycling of silicone materials, a class of polymers often mistakenly perceived as “non-recyclable”.

## 2. Applications of Silicones in the Building and Construction Industry and the Importance of Recycling for This Sector

The global building and construction (B&C) industry is one of the largest users of silicone products, with an estimated 673,000 metric tons sold annually [12]. Silicones have become the material of choice for protecting, reinforcing, insulating, and preserving buildings and structures, improving their energy efficiency and performance, and enabling new aesthetic and innovative features (see Table 1) [13,14,15].

The primary silicone products used in B&C are sealants, adhesives, gaskets, protective coatings (all of which can typically be classified as elastomers), and water repellants. Silicones are also widely used as additives or process aides in the manufacture of many of B&C’s key products (e.g., to stabilize polyurethane foams).

B&C is a vital industry that provides jobs and creates buildings and structures that connect communities and improve the quality of our lives. At the same time, this industry consumes more resources and produces more waste than any other sector of the economy. In Europe, it is responsible for about 40 percent of the CO_2_ emitted and one-third of the waste generated. Although the potential for recycling B&C materials is recognized, it is still underutilized.

Silicone can be recycled, but the recycling process requires specialized facilities equipped with the necessary technology and expertise to handle this material. Chemical recycling technologies offer the most promising solution. As we will discuss in more detail below, depolymerization of silicone allows for multiple recycling cycles without degradation in quality. In these processes, end-of-life (EoL) silicones are catalytically split into silicone oligomers and then repolymerized to virgin-quality silicone polymer.

Recycling silicones through depolymerization would not only reduce greenhouse gas emissions, conserve natural resources, reduce landfill waste, and reduce energy consumption, but it would also support the European Union’s goals under the Critical Raw Materials Act by reducing its dependence on imported silicon metal.

Silicone waste is generated during the manufacture of silicone products and their use in the construction and maintenance (renovation) of buildings but is also part of demolition waste. The fact that most silicone products used in the B&C industry are elastomers facilitates collection, but several key challenges remain: identifying silicones in unsorted waste, separating silicone products from packaging (e.g., plastic cartridges), developing facile depolymerization processes that are unaffected by the type of filler used in the silicone product, and improving the safety, efficiency, and selectivity of catalytic recycling processes on an industrial scale (multi-ton batch or continuous processes).

We will discuss these challenges and the progress that has been made in the search for new, cost-effective, closed-loop recycling processes below.

## 3. Challenges for Companies in the Silicone Recycling Business

Companies in the silicone recycling business face numerous challenges, most of which differ only gradually from those faced by the recycling industry in general [16,17]. The most important are the following:

- Diversity of industries, applications, and geographies;
- Diversity of product forms;
- Chemical complexity due to wide variety of formulations;
- Durability of silicone materials.

### 3.1. Diversity of Industries and Applications

Waste is generated throughout the life cycle of silicones, including production, use, and post-consumer disposal. The diversity of industries that use silicone products, from medical tubing to adhesives and sealants, and the complexity of their associated value chains create logistical challenges and add considerable costs to the recycling business, as specialized waste collection systems must be developed for each industry.

Circumstances vary greatly by industry and geography. While setting up a collection system (even across the entire value chain) may be relatively straightforward in some industries, such as automotive or electronics, it is a much more difficult task in others, such as building and construction. Furthermore, customized products (e.g., silicones used in food contact or medical applications) require the establishment of specific collection schemes.

A recent study, focused on developing economies, found that joint investment by different recycling companies may lead to a worse outcome than individual investment in a dedicated recycling infrastructure [18]. This seems to be particularly true for products with highly differentiated recycling costs.

### 3.2. Diversity of Product Forms

The wide variety of silicone uses and resulting product forms [19] pose a problem for recycling and a circular economy. In a considerable number of applications, silicones are used only in small quantities (as additives). Currently, separation and harvesting of the silicone in these applications is neither technically nor economically feasible. Examples of such applications include silicone emulsions used in hair care products that end up in wastewater treatment plants after use, or additives in paints and coatings that end up in landfills with construction waste.

**Figure 1:**
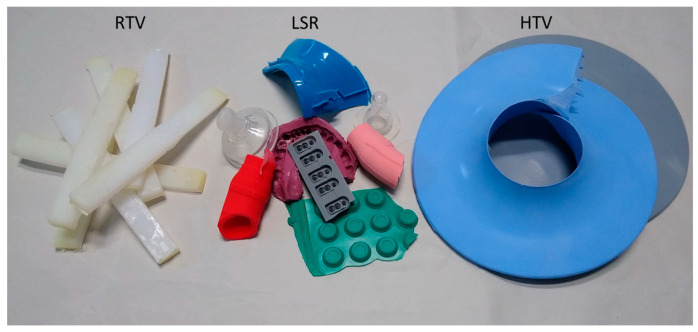
Post-industrial and post-consumer silicone elastomer waste (RTV: room temperature vulcanizate—typically sealants; LSR: liquid silicone rubber; HTV: high temperature vulcanizate) © Stammer Chemie GmbH.

As a result, elastomers make up most of the silicone waste available for recycling and a competitive recycling process must be able to recover these with the best possible yield and put them to a new, equivalent use. Typical examples of elastomers available for recycling include technical items such as silicone seals and sealants, tubing, gaskets, computer keyboards and covers, high-voltage insulators, as well as consumer silicone items such as kitchenware (baking pans, table mats) and personal accessories (pacifiers and teething rings, ear plugs, and wristbands). Some of these items are shown in Figure 1. While traditional plastics can be melted and reshaped due to their thermoplastic nature, making them easily recyclable, this method cannot be applied to elastomers in general (exemption to the rule are thermoplastic elastomers). Silicone elastomers are also more difficult to recycle than linear silicone polymers (as in silicone fluids and greases) due to the presence of crosslinking units.

### 3.3. Chemical Complexity Due to Large Variety of Formulations

Unlike thermoplastic polymer-based products, silicone elastomer formulations are highly complex, consisting of multiple components that synergistically contribute to the function and properties of the cured product. Without a selective sorting process, the wide variety of formulations results in a highly heterogeneous feed stream to the recycling process, even when only considering silicone elastomers as a product form. The highly variable composition of these materials in terms of catalysts, fillers, plasticizers, curing agents, and other additives, as well as contamination from the production, use, and disposal phases, can cause problems and even incompatibility during recycling.

### 3.4. Durability

While the inherent durability of silicones is a form of sustainability that directly benefits consumers in many applications—as more durable products offer longer life, better value, and reduced environmental impact—it is this very characteristic that makes chemical recycling more challenging [20]. The extreme stability and durability of the siloxane backbone means that silicones are difficult to depolymerize into monomers.

## 4. Dynamics of the Regulatory Framework

The problem of increasing resource consumption and associated waste volumes caused by our linear economic system has been discussed since the 1970s (Club of Rome). However, it is only recently that the public, politicians, and businesses have been calling for a circular economy, in which material resources are reused rather than endlessly disposed of. Plastics recycling is currently one of the focal points of policy efforts. This is reflected in the intense focus on the issue by political bodies at both international and national levels [21,22].

Three factors are driving political action: First, the rapid increase in plastic consumption: in the last two decades, from 2000 to 2020, the amount of plastic ever produced globally has more than doubled. Second, the disposal problem, especially for developed, high-income countries, has been exacerbated by the fact that exports of plastic waste to less developed countries have been drastically restricted worldwide (this development was led by China’s Operation National Sword (ONS) initiative from January 2018, followed by India with the Waste Import Ban in March 2019). Third, the public now perceives marine and land-based plastic pollution as a global threat to human and animal habitats.

At present, the closing of material cycles is supported by individual political initiatives at national and international levels. However, there is no institutional framework in Germany, the European Union, or elsewhere that fully supports a circular economy from a regulatory point of view [23]. As a result, the political, social, and regulatory environments are subject to strong dynamics.

The European Commission recently published a strategy that aims to reorganize the plastics economy by catalyzing the transition from a linear economy (take → make → waste) to a circular economy [24]. In this circular economy, the useful life of products should be maximized, and, at the end of their useful life, products should be available as secondary raw materials for new products. The goal is to minimize resource use and CO_2_ emissions throughout the extended product life cycle.

As a key pillar of the European Green Deal and the EU Taxonomy (alongside the key climate legislation), the Circular Economy Action Plan will be promoted and further developed in the coming years, for example, through measures such as setting collection and recycling quotas, increasing CO_2_ prices, subsidizing green investments, removing barriers such as the REACH classification of secondary raw materials, etc.

## 5. Value Chain of Silicones (PDMS)—Sources and Sinks

**Figure 2:**
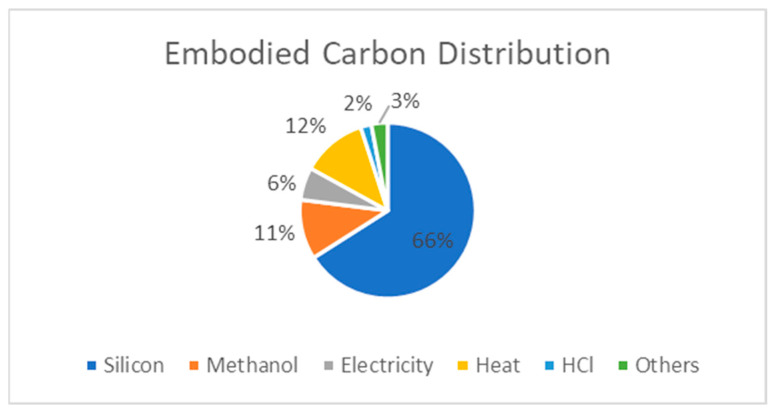
Typical embodied carbon resulting from the use of non-renewable energy sources in the production of silicone polymer [26] (redrawn from [27]).

**Figure 3:**
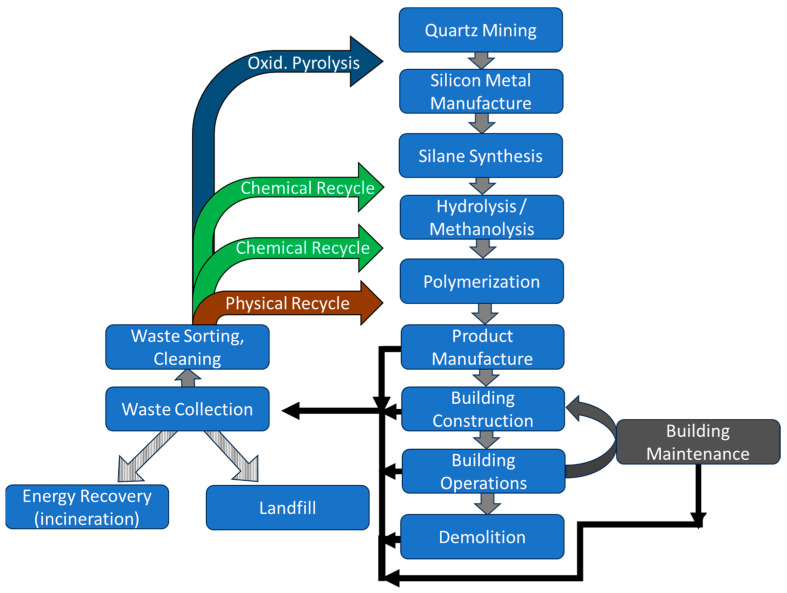
Silicone value chain steps, waste sources, and sinks in the construction and building industry—recycling options.

To understand the benefits of silicone recycling in terms of resource, energy, and capital efficiency, it is worthwhile taking a closer look at the conventional silicone manufacturing processes, as the traditional method of synthesizing silicone polymers requires substantial energy inputs and is capital-intensive [25] (see Figure 2 for the embodied carbon distribution in silicone polymer and Figure 3 for the value chain steps and recycling options).

There are seven key steps involved in producing virgin silicone:

(1) Production of silicon metal: Metallurgical silicon (about 98–99% pure) is produced from quartz (the most abundant mineral in the Earth’s crust) by reacting it with a carbonaceous material (coke, coal, or charcoal) as a reducing agent at temperatures of 1800–2000 °C in a submerged arc furnace (carbothermic reduction smelting). This is a very energy- and carbon-intensive process. Each ton of silicon produced requires 10–15 MWh of electricity and generates about 5 tons of CO_2_ [28]. Global metallurgical silicon production in 2022 was close to 4.5 million tons, of which about 35% was used to produce silicones and silanes [29]. Countries producing metallurgical silicon are China (77%), Brazil (7%), Norway (6%), France (4%), Russia (2%), Canada (1%), the USA (1%), and Germany (1%), with market shares shown in brackets [30]. Efforts are being made to reduce the environmental footprint of silicon production by investigating an aluminothermic reduction process that generates no direct CO_2_ emissions as an alternative to carbothermic reduction smelting [31].
(2) Production of methylchlorosilanes: On an industrial scale, the direct synthesis of chlorosilanes (Müller–Rochow process) is achieved by mixing finely ground metallurgical-grade silicon with a copper-based catalyst and minor amounts of zinc and various other promoter elements in a fluidized bed reactor and reacting this mixture with gaseous methyl chloride (CH_3_Cl) at a temperature of 280 to 350 °C and a pressure of 1 to 10 bar. This reaction is exothermic, and the yield is in the range of 85 to 90% [32,33].
(3) Separation of methylchlorosilanes: To separate the silanes and remove impurities, fractional distillation (rectification) is used. The most desirable product of direct synthesis is dimethyldichlorosilane, as it is the starting material for the synthesis of silicone polymers. An essential feature of dimethyldichlorosilane for its further use in the production of linear silicone polymers is an extremely low presence of trifunctional or tetrafunctional chlorosilanes, as these would lead to undesired crosslinking. Because the boiling points of the various silanes and impurities differ only slightly, the distillation of dimethyldichlorosilane, which is typically performed continuously, requires tall columns with very high separation efficiencies [34].
(4) Hydrolysis or methanolysis of dichlorodimethylsilane: The reaction yields a dimethylsilandiol, which is unstable and readily reacts by inter- or intramolecular condensation in the presence of HCl as a catalyst to an oligomeric mixture of cyclodiorganopolysiloxanes (“cyclics”) and hydroxyl-terminated dimethylsiloxanes (α,ω-polysiloxanediols, “linears”). The molar masses of both the linear and cyclic compounds are comparatively low.

**Figure 4:**
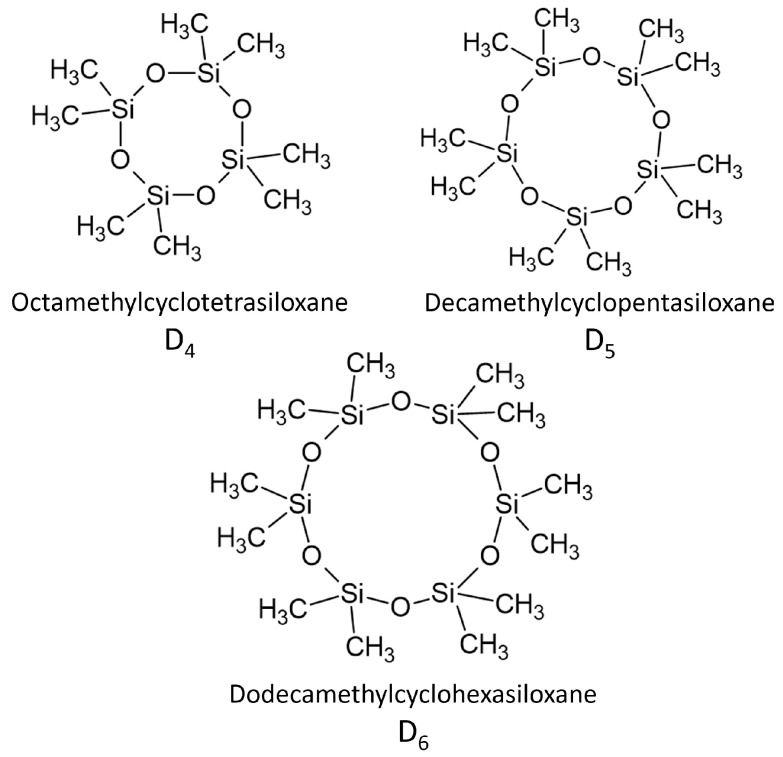
Cyclic siloxane oligomers (D_4_ to D_6_).

**Figure 5:**
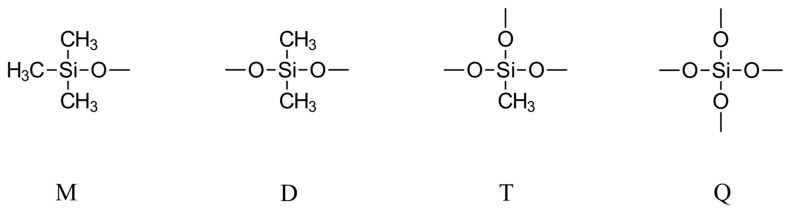
Structural elements M, D, T, and Q with methyl substitution.

The siloxane cycles, (R_2_SiO)_n_, are primarily six- to twelve-membered rings (D_3_ to D_6_) (see Figure 4), while the degree of polymerization (DP) of the linear siloxanes typically is between 30 and 80 (Note: In this paper, we follow the widely used silicone industry shorthand notation, introduced by Hurd in 1946 [35], in which different structural elements of siloxanes are assigned the symbols M, D, T, and Q, representing R_3_SiO(0.5), R_2_SiO(0.5)_2_, RSiO(0.5)_3_, and SiO(0.5)_4_, respectively (see Figure 5). This notation is based on the terms mono, di, tri, and quaternary, which denote the oxygen substitution on the silicon atom per structural unit).

By varying the reaction conditions, the product distribution between linear and cyclic compounds, as well as the molar masses of the linear and cyclic compounds, can be controlled over a relatively wide range [36]. Using the Wacker and Bayer processes, respectively, methanolysis allows for preferentially producing linear or cyclic oligomers [37]. The methyl chloride produced as a byproduct of the methanolysis reaction is fed directly back into the Müller–Rochow synthesis, while the hydrogen chloride produced in the hydrolysis process must first react with methanol in an additional esterification step.

(5) Separation of the cyclic and linear siloxane oligomers: After any trace of active catalyst has been quenched, the oligomers are washed with water and dried. The cyclosiloxane fraction can then be separated from the linear siloxanediols by flash distillation.
(6) Polycondensation of the linear siloxanediols or ring-opening polymerization of the cyclosiloxanes: Both polycondensation [38] and ring-opening reaction [39,40,41] are typically performed at elevated temperatures, are catalyzed by many acids or bases, and yield a mixture of cyclic oligomers along with a polydisperse distribution of polymers. Depending on the process and conditions selected, contamination of the silicone polymer by volatile cyclosiloxanes is either present at levels low enough to not warrant removal or, if present at higher levels, is removed by vacuum stripping [42]. Polycondensation, especially in the presence of a phosphonitrilic chloride catalyst [43], is preferred because of its high reaction rate and the resulting very low contamination with oligomeric cyclic siloxanes. Upon completion of the polymerization or polycondensation reaction, the catalyst must be effectively quenched, because catalysts used in the preparation of silicones can also catalyze their depolymerization, especially when silicones are exposed to high temperatures [44].
(7) Compounding the silicone product: In general, unmodified polysiloxane networks have poor mechanical properties and limited adhesion. Therefore, in addition to polysiloxanes, silicone products typically contain reinforcing fillers, plasticizers, crosslinkers, adhesion promoters, and other additives to achieve the desired processing and performance characteristics. These ingredients are incorporated into the silicone polymer by compounding (blending) in a batch or continuous process. Once cured (crosslinked), the filler forms a heterogeneous secondary phase that reinforces the polymer matrix [45,46,47,48]. Therefore, at the molecular level, commercial silicone elastomers can be understood as a complex, heterogeneous composite material.

## 6. Silicone Recycling Methods

Silicone elastomers are thermosets, which do not melt like thermoplastics. This makes them much more difficult to recycle and reuse. There are two primary methods of recycling silicone thermosets: mechanical recycling and chemical recycling.

### 6.1. Mechanical (Physical) Recycling

**Figure 6:**
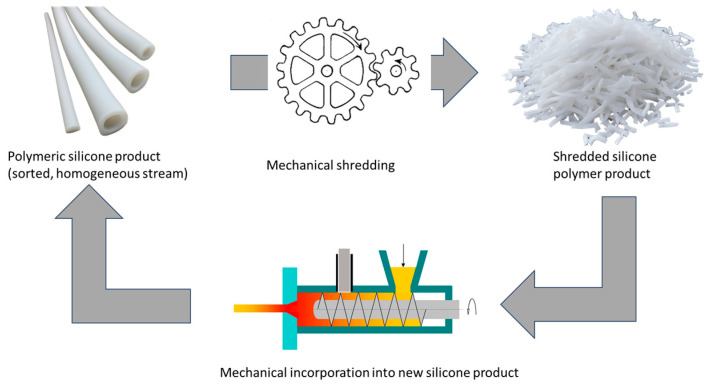
Schematic representation of the mechanical recycling of silicone rubber.

Mechanical recycling involves the processing of silicone elastomer waste through a series of shredding, grinding, crushing, and (cryogenic) milling steps that convert the silicone waste into finely ground (micronized) particles that can be used as an additive in a variety of formulations, including sealants, adhesives, and coatings (see Figure 6).

From an environmental perspective, physical recycling is generally considered the preferred method due to its high efficiency and (relatively) low energy consumption. However, physical recycling has its own limitations. A key limitation is that physical recycling can only cover certain (homogeneous) waste streams and requires extensive pre-sorting and cleaning. The purity of the recycled material is highly dependent on the specification of the waste. Contaminated waste can have a highly negative impact on the quality of the new product, as physical recycling typically involves mixing the recycled material with virgin material. With each reuse of the silicone material, contaminants and problematic chemicals from previous uses are thus spread over an ever-increasing volume.

Researchers, studying the effect of incorporating silicone rubber (HTV) powder, obtained by mechanical grinding, into virgin silicone rubber (HTV) found that the recycled powder did not alter the glass transition and crystallization temperatures, but decreased the tensile and tear strength by about 20%, and modulus by 15%, while the hardness underwent only marginal changes [49]. With repeated mechanical recycling, silicone elastomers tend to degrade continuously in the process and are unable to maintain their quality after two or more recycling loops. Because mechanical recycling produces material of lower quality than virgin silicone and cannot be repeated indefinitely, it is typically referred to as downcycling.

### 6.2. Chemical Recycling

**Figure 7:**
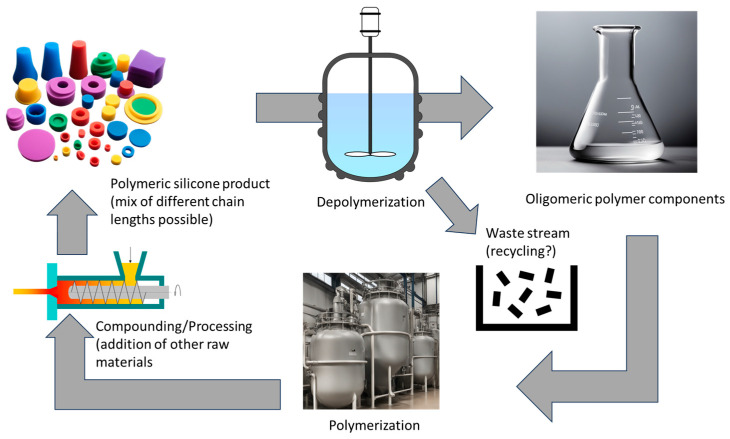
Schematic representation of the chemical recycling of silicone rubber.

Chemical recycling, also known as feedstock recycling, involves mechanisms that use heat, chemical degradation agents, or both, typically in the presence of a catalyst, to depolymerize silicone waste into oligomers (see Figure 7). A characteristic of silicones is the formation of thermodynamically controlled ring–chain equilibria during both their preparation and their thermal degradation [50,51]. This property is exploited in the chemical recycling of silicones, for example, by heating the silicone waste under acid or base catalysis and continuously removing the cyclosiloxanes from the equilibrium. As polydimethylsiloxane is the most widely used silicone due to its versatile properties, most of the recycling processes are based on depolymerization of this polymer.

Compared to mechanical recycling, a disadvantage is the higher energy input required, but there is a significant advantage: chemical recycling is a closed-loop process that promises endless virgin-grade recycling because these oligomers can be repolymerized by seamlessly integrating them back into the basic silicone manufacturing process.

In addition, chemical recycling of silicones offers two major advantages over mechanical recycling:

- It can be applied to mixed, heterogeneous waste streams and does not require the same level of careful sorting and cleaning because it allows for the removal of contaminants.
- It enables product upcycling—the transformation of silicone waste into higher-value products (as defined in [52])—because there are no restrictions on the new, value-added products that can be created through chemical recycling.

**Figure 8:**
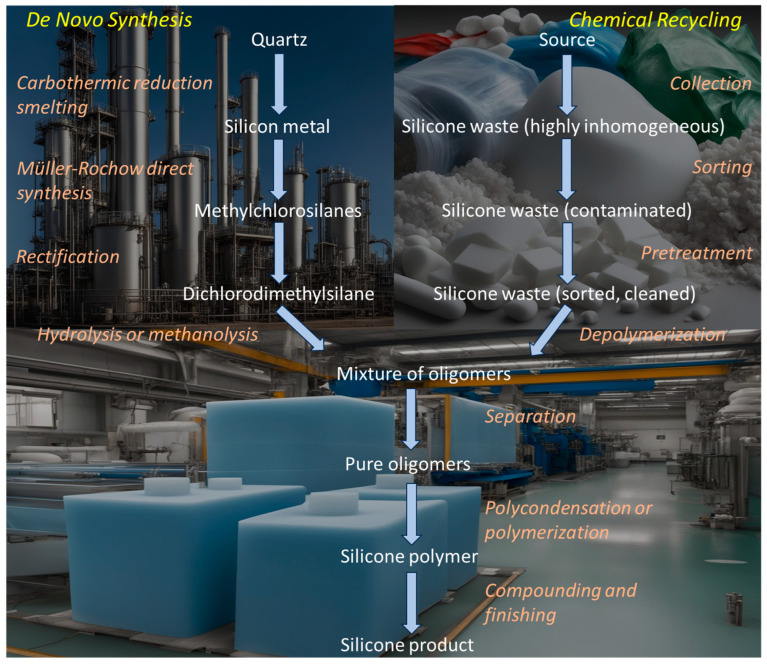
Process steps in the manufacturing of virgin and chemically recycled silicone products.

Chemical recycling of silicones replaces the economic and environmental costs of producing virgin oligomeric material (steps 1–4 in the silicone value chain) with the corresponding costs of the recycling process steps (collection, sorting, pretreatment, and depolymerization) (see Figure 8).

The benefits of chemical recycling of silicones are qualitatively obvious: recycling helps reduce waste and CO_2_ emissions and improves the resource intensity and energy efficiency of silicone production. However, quantitative data are scarce.

According to the silicone recycling company ECO U.S.A., recycling 1 ton of silicone rubber using their proprietary process saves 5774 kWh of energy, 16.3 barrels of crude oil, and about 2587 m^3^ of gas (equivalent to 28,722 kWh of heating energy) [53]. Life cycle analyses (LCAs) performed by the same company according to ISO 14067 [54] (cradle to gate) showed that the greenhouse gas emissions resulting from the production of 1 ton of virgin silicone oil (non-reactive polymer) are 6079 kg CO_2_eq, while this amount is only 1401 kg CO_2_eq for silicone oil obtained from the recycling process [55].

Dow Chemical expects that recycling silicone waste streams will enable a significant reduction of over 50% in the carbon footprint of PDMS production compared to virgin production [56]. This approach avoids the need to produce silicon metal, which accounts for 55–65% of traditional silicone production. Additionally, recycling can minimize direct (Scope 1) and indirect (Scope 3) emissions. Direct emissions are directly attributable to a company’s operation, while indirect emissions result from the incineration or landfilling of silicone materials at their end-of-life. Dow states that this estimate is subject to a pending independent third-party review of the life cycle analysis, conducted in accordance with ISO 14040 [57] and ISO14044 [58] standards.

However, the chemical recycling of silicones does not come without specific challenges:

(a) After depolymerization, large quantities (typically 10–50% by weight for elastomers and sealants) of micro-sized, solid components (fillers, pigments, and additives) must be removed, for instance, by passing the liquid through highly efficient filters.
(b) In addition, intermediates containing monovalent (M) units (end groups) or trivalent (T) units (branching points) must be quantitatively separated from those based on divalent (D) units (chain segments) because any carryover of even small amounts of M and T units (or T’ from peroxide or addition cures) into the hydrolysate (linear or cyclic oligomers) will render it unsuitable for subsequent polymerization.
(c) Catalysts and auxiliary additives, such as surfactants and solvents, must be effectively separated from the reaction mixture.
(d) Gaseous impurities that are formed during the depolymerization process must be captured and removed.
(e) Depending on the chosen degradation chemistry, certain reactive groups found in some silicone products can interfere with chemical depolymerization. For example, amines found in many textile and cosmetic products and some sealants can interfere with acid degradation. Fillers such as chalk that react with acids can also have a negative effect on the depolymerization process.
(f) As with the hydrolysis or methanolysis of chlorosilanes, silicone recycling produces not just one precursor (“monomer”) for subsequent polymerization, but many. Controlling the proportions of linear and cyclic components and their composition (chain length for linear components, ring size for cyclic components) is an additional challenge to recycling efficiency.

## 7. Characteristics of an Ideal Process for the Chemical Recycling of Silicone

Ideally, the depolymerization process should meet the following criteria:

(a) Economics:
  - High yield;
  - Easy scalability from laboratory to pilot to commercial scale.
  - High quality output (product is free of SiH groups, multifunctional (T and Q) units, and hexamethyldisiloxane (MM)).
  - High degree of adaptability (in terms of the types of silicones present in the waste).
  - Low cost of reactor components (reaction components are non-corrosive or only slightly corrosive).
  - Low cost of catalyst materials (in terms of purchase price, reuse, and quantity used).
(b) Environmental impact and safety:
  - Maximum recyclability (ability to recycle most byproducts, such as fillers or catalysts, in separate processes), resulting in less waste.
  - Good environmental compatibility and low health risk of the chemicals used and the resulting wastes (not corrosive, toxic, or highly flammable).
  - Mild reaction conditions (ideally at room temperature and normal pressure), but fast conversion (low energy input).

None of the currently used chemical recycling processes meet all of the above criteria.

## 8. Silicone Depolymerization Mechanisms

Research into the effects of heat, radiation, and other degradation factors on the stability of silicones has been conducted since the early days of the industry [59,60] and continues to this day [61]. The primary goal of these studies was to improve the durability of silicones when exposed to various environments. This research has provided valuable insight into specific degradation mechanisms, which were further evaluated in model studies, frequently using linear siloxane polymers. The key finding of these studies was that polysiloxane degradation is influenced by two factors: the type and concentration of polymer end groups and the presence of catalytic amounts of impurities in the polymer.

Polysiloxanes containing ionic or polar impurities or additives, even in very small amounts, can thermally degrade by an “externally catalyzed” mechanism. In this mechanism, heterolytic cleavage of the siloxane backbone is initiated by a non-silicone species, such as trace amounts of residual acidic or basic polymerization initiators that have not been effectively quenched. These impurities are often the Achilles’ heel of cured silicone elastomers in terms of their heat stability.

Further studies were motivated by the development of “silicone strippers” that could “digest” excess cured silicone sealants by breaking down the polymers into smaller molecules that could be more easily removed from the contaminated surface (see, for example, [62,63]).

Linear polysiloxanes decompose into a mixture of low molecular weight cyclic oligomers when heated to their decomposition temperature in an inert atmosphere. For PDMS, the cyclic oligomers [(CH_3_)_2_SiO]_n_ have a degree of polymerization (n) ranging from 3 to as high as 20 and are volatile at the decomposition temperature. The diffusion of volatile cyclic oligomers from the bulk silicone material becomes the primary limiting factor in the rate of PDMS degradation [64]. The driving force for the degradation process is the higher, entropy-driven thermodynamic stability of the small cyclodimethylsiloxane oligomers compared to the linear, open-chain polymers at the degradation temperature [65].

**Figure 9:**
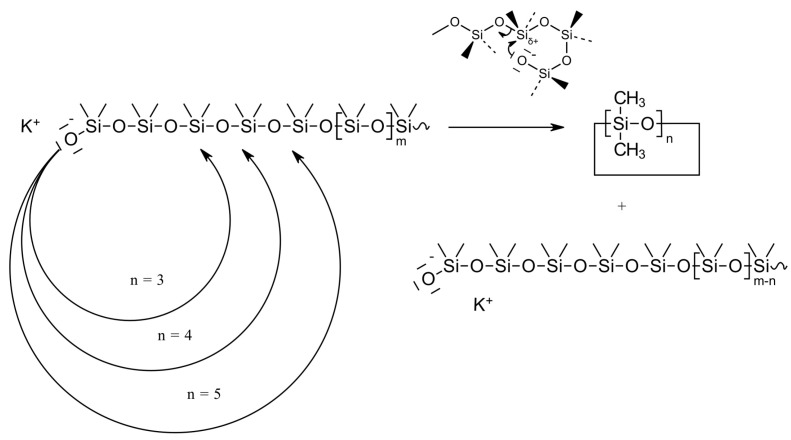
Schematic of the externally catalyzed mechanism with the transition complex shown for hexamethylcyclotrisiloxane (D_3_).

Polysiloxanes may degrade by three different reaction mechanisms, called “unzipping” [66,67], “random scission” [68], and “externally catalyzed” mechanisms [69] (see Figure 9).

Polysiloxanes containing either silanol (Si-OH) or hydroxyalkyl (Si-R-OH) end groups undergo depolymerization by an “unzipping” degradation mechanism. In the unzipping mechanism, hydroxyl groups at the polymer chain ends can “back-bite” to promote intramolecular redistribution reactions [65,70,71]. Due to their higher stability at the degradation temperature, these reactions predominantly produce the cyclic trimer and tetramer. Volatilization of the cyclic oligomers results in a shortening of the siloxane polymer, with the decrease in polymer molecular weight being linear with the degree of volatilization. This is consistent with the stepwise elimination of volatiles and is a characteristic of the unzipping reaction mechanism in general. Depolymerization from the chain-ends continues until the polymer molecule is completely degraded and no non-volatile residue remains at the end of the process.

Depolymerization of polysiloxanes with endcapped inert groups can also occur randomly—at any point in the polymer chain where a temporary loop may be formed—by intramolecular or intermolecular redistribution reactions at or between the siloxane backbones. In the intramolecular “back-biting” mechanism, the polymer chain folds back on itself in a cyclic transition state [68], where a rearrangement of the siloxane bond occurs, resulting in the cleavage of a cyclic siloxane oligomer [60,72]. The back-biting continues until the structure is too short to cyclize and/or evaporation of the shortened bistrimethylsilyl-terminated chain component occurs. This “random scission” process predominates at high temperatures and results in a much faster decrease in polymer molecular weight than the unzipping mechanism, while simultaneously broadening the molecular weight distribution. Both effects are caused by the fact that the most likely site for random scission is near the center of the polymer chains. Random scission is the most common degradation mechanism of polysiloxanes, regardless of their polymeric structure and the actual degree of purity of the polymer.

In linear siloxane polymers, cleavage of the Si-O bond at one site is sufficient to initiate degradation. However, when the siloxane polymer is crosslinked into a network structure, cleavage at two or more sites is essential for degradation [73].

The above mechanisms all involve the formation of intermediate four-center transition states and the elimination of volatile cyclic siloxanes. The prerequisites for the cyclosiloxane elimination mechanisms are the high flexibility of the siloxane chain segments, which allows for relatively easy formation of the transition state, and the susceptibility of the siloxane bond to heterolytic cleavage. While the Si-O bond (451 kJ/mol) is stronger than the Si-C bond (326 kJ/mol) in terms of its homolytic dissociation energy, the heterolytic cleavage of the Si-O bond is energetically favored by the overlap of empty silicon d-orbitals with occupied orbitals of oxygen atoms involved in the cyclic transition state [74].

In the presence of oxygen, siloxane degradation is more complex (as studied by Camino [64] on linear PDMS). At temperatures below about 300 °C, oxygen acts as a catalyst and promotes the cleavage and rearrangement of the Si-O bonds. The removal of the volatile oligomers therefore occurs at a much lower temperature than in an inert atmosphere. At higher temperatures, however, the remaining siloxane polymer undergoes oxidative crosslinking, which reduces the rate of mass loss in the final stage of degradation. Consequently, in an oxidative atmosphere, a silica residue (approximately 10% of the mass of unfilled silicone) is formed due to the competition between oxidative crosslinking and volatilization of oligomers [75].

## 9. Depolymerization Chemistries

Most of the research on thermal and/or catalytic degradation of polysiloxanes to date has focused on improving the understanding of the mechanisms involved rather than on developing depolymerization methods for actual industrial silicone products. Consequently, while the general mechanisms have been established, little is known about the effects of additives, fillers, and different network topologies on the degradation chemistry and overall composition of the resulting products.

The depolymerization mechanisms that occur in crosslinked siloxane systems, such as elastomers or sealants, are influenced by the network architecture (crosslink density [76]; combination of M, D, T, and Q units; chain length between crosslinks; degree and type of free chain ends [70]; type of chemical substituents along the polymer backbone) [70]; formulation ingredients (catalyst and filler types and levels); and degradation process parameters (temperature, type of ambient atmosphere, and sample mass and geometry) [77,78].

The partially ionic nature of the siloxane bond results in high reactivity with electrophilic and nucleophilic agents. As a result, silicones undergo thermal catalytic or thermal depolymerization at temperatures ranging from ambient to about 550 °C. Within the lower temperature range, depolymerization reactions occur primarily along the Si-O-Si backbone, while at temperatures above 350 °C, degradation takes place increasingly at the Si-C bonds, leading to elimination of the substituent groups, and, at temperatures above 550 °C to calcination (mineralization) of the silicone. The temperature range useful for depolymerization can be roughly divided into three regimes:

(a) Low temperature (20–180 °C): depolymerization in the presence of strong Brønsted acids or bases;
(b) Medium temperature (180–350 °C): depolymerization induced by “weaker” nucleophiles (Lewis bases) or electrophiles (Lewis acids);
(c) High temperature (350–550 °C): thermally induced depolymerization.

**Figure 10:**
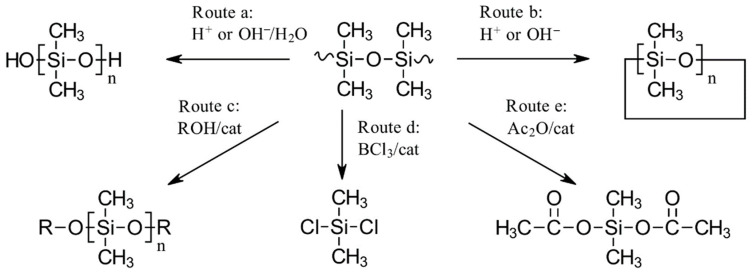
Some examples of depolymerization routes currently practiced or evaluated by industry.

To date, scission of the siloxane bonds as the first step leading to the depolymerization of silicone polymers has been explored using many different degradation chemistries. We refer the interested reader to the recent publications by Rupasinghe [7,8], Rupasinghe and Furgal [9], Muzafarov et al. [10], and Elmanovich et al. [11], which discuss these options in more detail. The latter publication also suggests using supercritical fluids to enhance these depolymerization processes. In our discussion, we will focus primarily on degradation chemistries that are, or have the potential to be, used in industrial-scale chemical recycling of silicones (while providing some historical background on their development) (see Figure 10 and Table 2).

As will be discussed in more detail below, of the depolymerization approaches shown in Figure 10, route a is likely to be the most efficient, if the formation of cyclic siloxanes (cyclomers) can be minimized and the resulting linear oligomers can be used without further purification. However, in current processes, significant amounts of cyclomers are formed and must later be removed due to regulatory constraints on cyclomer content in polymers.

Route b is currently the most widely used on an industrial scale. Routes c-e are either in use today on a smaller scale or an industrial process is under development. Routes a and b have the lowest risk of introducing impurities into the final siloxane polymer if carried out in an alkaline medium; however, the purity of the silicones obtained will depend mainly on the efficiency of the physical purification processes used after the intermediates obtained by the different routes have been converted into linear or cyclic siloxanes.

Table 2 provides only basic information on the methods; details and alternative catalysts are described below. While fluids and polymers typically do not require solvents for any of the methods, solvents are often used when recycling elastomeric materials. Solvents are then selected to be easily separated from the resulting intermediates, or low molecular weight siloxanes can be used as an alternative.

### 9.1. Depolymerization by Acidolysis with Protonic Acids

In the acidolysis reaction, the hydronium cation [H_3_O(H_2_O)_3_]^+^ provided by the acid attaches to the electron-rich oxygen atom. This dramatically reduces the energy barrier of the Si-O cleavage reaction. In addition, the proton donor–acceptor characteristics of the medium, as well as the presence of water or other additives capable of forming strong hydrogen bonds, strongly influence the rate of the cleavage reaction. When water subsequently attacks the silicon atom in a nucleophilic reaction, two silanol bonds are formed, which can be further rearranged by backbiting reactions to form cyclic siloxanes or linear siloxane diols [79].

As early as in 1946, Patnode and Wilcock reported [70] on the ring-opening polymerization of cyclic oligomers induced by sulfuric acid at room temperature; therefore, it was only natural to consider acidolysis for the depolymerization of PDMS.

In 1954, Humphrey and Wasserman [80] demonstrated that anhydrous acids such as gaseous hydrochloric acid were capable of partially depolymerizing silicone rubbers at ambient temperature. Interestingly, they also found that glycerol, when added to the liquefied rubber, stabilized it in its liquid form.

Solvents that solubilize the acid catalyst and swell the PDMS network promote depolymerization and facilitate the separation of fillers. This was observed by employees of Midland Silicones (Dow Corning) who reported in 1954 the rapid decomposition of a HTV silicone when exposed to dry hydrochloric acid bubbled through a hydrocarbon or chlorohydrocarbon solvent [81].

In 1981, Burkhardt and Louis [82] described a process for preparing cyclic siloxanes by reacting linear polydimethylsiloxanes with 50–85% aqueous sulfuric acid at 130–150 °C for 1.5 to 6 h. The cyclic species were recovered by distillation with a yield of 89%.

In 1992, Greenlee [83] disclosed a process that depolymerizes high molecular weight silicone polymers by dissolving them in a high-boiling organic solvent (diethylene glycol monobutyl ether) containing sulfuric acid at 150–180 °C, followed by reducing the temperature of the solution to about 80–115 °C, adding an excess of potassium hydroxide to drive the reaction to completion, and distilling the cyclic products from the solution.

While the addition of solvents can improve the yield, it requires an additional processing step to remove the solvents from the cyclic oligomers and regenerate them for later use. Such additional processing steps result in higher energy consumption, making the recycling process less economical. Other disadvantages of a solvent-based process include environmental issues (VOCs) as well as hygiene and safety concerns. Therefore, it is more desirable to depolymerize under solvent-free conditions. Furthermore, a solid catalyst would be preferable to simplify the sulfuric acid separation process and reduce waste.

To address the shortcomings of the currently used processing technology, inventors from Hefei University of Technology, China [84], propose a solvent-free process in which sulfuric acid is immobilized on an inorganic sintered Al_2_O_3_/TiO_2_ support. Using this catalyst at a level of 0.5–5% (by weight) in the degradation process, keypad scrap silicone rubber is depolymerized at 120–150 °C within 3–4 h. The use of solid acidic catalysts, such as sulfuric acid-treated clays [85] or acidic zeolites [86], had been reported previously, but these catalysts required much higher temperatures to be effective, typically above 300 °C.

Oligomeric siloxanes, such as decamethylcyclopentasiloxane (D_5_), can also be used to swell cured silicone before or during depolymerization. For example, Knott and Dudzik depolymerized a cured sealant using either concentrated sulfuric acid or concentrated trifluoromethanesulfonic acid and, by adding a suitable endblocker, were able to repolymerize the mixture to form functional endcapped polymers by heating the mixture to 50–120 °C for 4 hours [87]. Another important benefit of the process is that it enables single-stream recycling of silicone-contaminated polyolefin cartridges by completely separating the cured silicone residues that typically adhere stubbornly to the wall, plunger, and applicator tip of used cartridges.

Rapid acidolysis of calcium carbonate-filled silicone sealants with strong acids can cause severe foaming. The foam can accumulate in the headspace of the reactor vessel, making it difficult to control and potentially leading to overflow, which can negatively impact production. To mitigate this problem, the fill level must be reduced, resulting in smaller batch sizes and reduced productivity. In addition, excessive amounts of acid are required to compensate for the calcium carbonate filler. The use of sulfuric acid can lead to the formation of calcium sulfate deposits in equipment, requiring their subsequent removal.

Neutral cure sealants (alkoxy and oxime cure) typically contain aminosilanes as adhesion promoters. In some acidolysis processes, the amount of acid used is low. For these processes, the amount of acid may need to be adjusted, as the primary or secondary amine groups present in these elastomers may otherwise interfere with the acid-catalyzed recycling process.

### 9.2. Depolymerization by Strong Inorganic Bases

In 1942, J. Frank Hyde heated polysiloxanes to about 300 °C to produce the cyclic tri- and tetramer. However, he also observed that monomers based on T and Q units were transferred with the distillate and that this could be prevented by adding NaOH to the decomposing polymer [88]. In 1946, he and his collaborators published a paper showing the advantages of using alkali or alkaline earth hydroxides, alkali metal fluorides, or mixtures of these hydroxides and fluorides as catalysts for depolymerizing polydiorganosiloxanes [89].

The depolymerization of silicone rubber waste in highly alkaline environments at lower temperatures, typically in the range of 80–120 °C, often in the presence of alkali fluoride co-catalysts, has been pursued primarily in Russia. As early as 1980, Andrianov and his coworkers demonstrated the suitability of this co-catalyst system for cleaving the Si-O bond in organosiloxanes and for depolymerizing silicone rubber waste at temperatures of 110–120 °C [90]. The use of the co-catalyst allows for the process to be run at a temperature about 20–30 °C lower than when using the KOH catalyst alone, while reducing the rate of silicon–carbon bond cleavage, thereby increasing the yield and quality of cyclic target products. Shapatin and his colleagues describe the depolymerization of silicone waste using alkali metal hydroxide or fluoride by passing steam and inert gas through the reaction mixture at 150–200 °C [91]. Alternatively, silicone rubber mixtures can be depolymerized at 140–190 °C with aqueous alkali metal hydroxide in the presence of a mixture of aluminum hydroxide and dimethylformamide as catalyst [92].

Voyloshnikov discusses the possibility of depolymerizing silicone rubber waste in an inert atmosphere or in a vacuum with KOH in the presence of a silicone surfactant as stabilizer at a temperature of 50–175 °C for 6–8 h [93].

While the use of inorganic fluorides as initiators allows for low process temperatures, it is not without its drawbacks. The fluoride anion is known for its ability to nucleophilically attack the silicon atom. Thus, Allandrieu and Cardinaud, who demonstrated the depolymerization of silanol-terminated linear PDMS with potassium hydroxide or quaternary ammonium hydroxide in the presence of catalytic amounts of quaternary phosphonium halides at 130 °C [94], point out that the use of fluorides can lead to compounds containing a fluorine-silicon bond, which limits the cyclization yield.

In the depolymerization of crosslinked siloxanes containing trifunctional (T) or tetrafunctional (Q) units, it is known that these units react with KOH to form non-volatile potassium salts. Thus, these units remain in the reaction mass and accumulate over time during the depolymerization process. In addition, demethylation of D-units induced by the alkali hydroxide catalyst at higher temperatures may result in the subsequent formation of further T (or even Q) units. As these units accumulate, they increase the viscosity of the reaction mass until a gel or residue is formed that must be discarded, usually by disposal in a hazardous waste landfill. Razzano teaches a method for making cyclic siloxanes in which an effective amount of high-boiling alcohol is used to prevent gel formation [95]. The preferred alcohols are those with boiling points above 300 °C so that they do not distill over with the cyclic siloxanes.

Yang and co-inventors describe an alkaline depolymerization process for silicone polymers and crosslinked rubbers using secondary and tertiary aliphatic alcohols in addition to NaOH or KOH as decomposition promoters [96]. According to these inventors, the process can be carried out at low temperatures, including ambient temperatures, and is more controllable than when primary alcohols are used as decomposition promoters.

Monteil and a team of researchers from the University of Lyon and Elkem Silicones [97,98] developed a method for recycling silicones using a highly effective silanolate catalyst obtained by chelating the potassium cation with a crown ether or its cheaper linear counterpart, polyethylene glycol dimethyl ether (Glyme). The weakly coordinating cation (WCC) increases the availability (dissociation) of the silanolate anion, making it highly nucleophilic. The depolymerization of linear polysiloxanes requires only a small amount of catalyst (typically 0.1 mol%) and the process can be operated over a wide temperature range (60–170 °C) to efficiently produce a mixture of cyclomers. Monteil and his team also demonstrated the depolymerization of a two-part, weakly crosslinked, addition cure RTV silicone gel with a 95% yield of cyclosiloxanes. Although not stated by the authors, we assume that a silicone gel (or any crosslinked silicone) must be silica-free to be recyclable; otherwise, the chelating agents may be deactivated due to their higher affinity for silica than for potassium.

Recently, Hoge and colleagues reported the first example of isolated silanol-silanolated anions [Si-O⋯H-O-Si] that are free of any contact with their weakly coordinating phosphazenium counter-cations [99,100,101]. Since these anions are strong nucleophiles, they could potentially be used as catalysts for the depolymerization of silicones. However, the authors obtained poor yields of cyclosiloxanes in the depolymerization of linear polysiloxanes, even when the reaction was carried out at 90 °C in a vacuum (7 mbar). In addition, these silanolate salts decompose rapidly above 100 °C in a vacuum, which restricts their use as catalysts for chemical recycling.

Miller and colleagues recently reported an entropy-driven depolymerization of silicones at ambient pressure using hexylene glycol (2-methyl-2,4-pentanediol) and NaOH as a catalyst at 175 °C [102]. Upon deprotonation by the base, hexylene glycol becomes a strong nucleophile capable of attacking silicon atoms both at the chain ends and in the main chain. Each step of the overall depolymerization reaction is an equilibrium reaction, therefore the product must be removed by distillation to avoid repolymerization.

### 9.3. Depolymerization by Aminolysis

**Figure 11:**
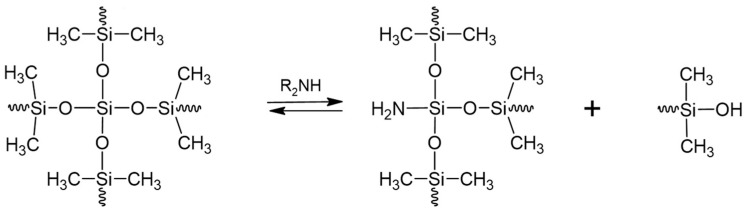
Cleavage of Q-unit by aminolysis.

In the mid-1970s, Hsiao and his colleagues discovered that aliphatic primary and secondary amines, when applied in super-stoichiometric amounts, were able to completely liquefy crosslinked silicones at room temperature by forming linear polymers [103], while tertiary amines, contrary to what was stated in Doyle’s 1951 patent application [104], were not able to do so. Since this is an equilibrium reaction, renewed polymerization and crosslinking can be achieved simply by removing the amine by distillation (see Figure 11). The dissolution is caused by the amines inducing a nucleophilic cleavage of the Si-O bonds, which occurs preferentially at the crosslinking sites due to the increased electrophilicity of the silicon atoms that are bonded to three or four oxygen atoms.

Schimmel [105,106,107], who studied the effect of adding potassium hydroxide as a second nucleophile to diethylamine (DEA), first on model siloxanes and later on polysiloxanes, found that KOH could accelerate the rate of aminolysis of silicone rubbers. Whereas diethylamine tends to cleave the silicone at the crosslinks, the addition of KOH is believed to increase the activity to the extent that cleaving of the siloxane chain units is feasible. However, the role of the KOH as a catalyst is not fully clarified.

In the late 1990s, Chang and his colleagues began to study the DEA/KOH aminolysis reaction in more detail [108]. The team observed that adding ethanol to the KOH/DEA solution dramatically increased the rate of aminolysis of crosslinked RTV-PDMS networks at room temperature, reducing the dissolution time from days to hours. This result was attributed to complete dissolution of the potassium hydroxide and easier diffusion into the silicone material at the liquid–solid interface. The product of the degradation process was composed of 85% cyclic oligomers.

A few years later, Chang and his team studied the effects of different swelling solvents on the aminolysis reaction [109,110]. In polar solvents, such as tetrahydrofuran (THF), the main degradation product was cyclic siloxanes, mostly D_4_, while in less polar solvents, such as toluene, the main products of the aminolysis reaction were linear dimethylsiloxane polymers (Mw of 15,000) with mixed hydroxyl and ethoxy end groups (EtO–PDMS–OH).

Huang, Ikeda, and Oku consider the removal of fillers as the main problem in the recycling of silicone rubber waste. In a previous study [111], they had observed that KOH with toluene as the swelling solvent was effective in degrading unfilled silicone rubber and that there was a non-linear relationship between the amount of potassium hydroxide used and the yield of cyclic siloxanes. However, HTV silicone rubber containing silica and alumina did not degrade even when excessive amounts of KOH were used and the material was refluxed in toluene for 10 h. In a more recent study [112], they found that a mixture of diethylamine, methanol, and hexane was extremely effective not only in facilitating the KOH-catalyzed depolymerization of filled silicone rubber, but also in enabling the separation of fillers by filtration prior to the removal of cyclic oligomers and solvent. By using an organic quaternary ammonium hydroxide instead of KOH [113], the filler was recovered with an even higher efficiency of 83–93% (*w*/*w*). This process was subsequently patented by Kyoto Institute of Technology and Kansai Electric Power Company [114,115].

A similar—but two-step—process was previously patented by Heidingfeldova [116], in which the silicone waste is first treated with DEA and tetramethylammonium hydroxide; then, the DEA is removed by vacuum distillation, a dispersion of KOH in D_4_ is added, the temperature is raised to 165 °C, and cyclic oligomers, primarily D_3_ to D_5_, are distilled under vacuum.

### 9.4. Depolymerization by Alcoholysis

**Figure 12:**
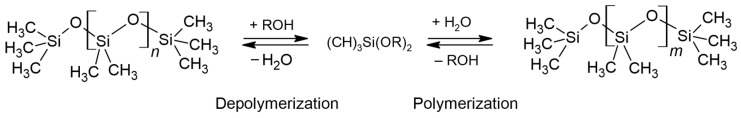
Recycling by alcoholysis reaction (schematic representation).

Alcoholysis of siloxane bonds is an equilibrium reaction; therefore, removal of water as a byproduct is essential to drive the reaction to completion (see Figure 12).

In 1959, Voronkov and Shabarova published the results of their experiments on nucleophilic cleavage of short-chain siloxanes by amyl alcohol in the presence of potassium hydroxide as a method to synthesize organoalkoxysilanes [117].

In the mid-1990s, Hirose, Tsuji, and Ouchi successfully achieved the partial depolymerization of silicone rubber using methanol, sulfuric acid, and trimethyl orthoformate (trimethoxymethane) as a water scavenger [118]. The water reacted with the orthoester to produce additional methanol. Gas chromatographic analysis of the depolymerized silicone confirmed the formation of dimethyldimethoxysilane.

In the early 2000s, Okamoto’s team was the first to use dimethyl carbonate as an environmentally benign, non-corrosive solvent and depolymerization agent to achieve deoligomerization of cyclic siloxane species using an alumina-supported potassium fluoride catalyst [119]. While the catalyst was deactivated during the deoligomerization of larger species, the team observed that the addition of a small amount of methanol suppressed the deactivation. Therefore, they speculated that this observation may have opened a new avenue for the recycling of silicones.

Shortly thereafter, they demonstrated that by adding methanol, longer-chain phenyl- and a vinyl-substituted polysiloxane oils and crosslinked rubbers could be depolymerized in a pressure reactor at 180 °C using potassium fluoride as catalyst in dimethyl carbonate [120]. The team showed that the siloxane bond is cleaved by methanol attack, as the depolymerization rate depended on the type of alcohol, but not on the dialkyl carbonate. Kawamoto was able to further reduce the reaction temperature to 90 °C by replacing the fluoride catalyst with 0.2 mmol of sulfuric acid, but he obtained a wild mixture of monomeric and oligomeric products of more than 200 different compounds, including dimethoxydimethylsilane, among others [121]. The non-selectivity of the otherwise mild depolymerization process makes it unattractive because any reuse of the material resulting from the decomposition reaction requires a complex thermal separation process.

The latter two reactions are likely to be viewed as alcoholysis with dimethyl carbonate acting primarily as a water scavenger by reacting to form methanol and carbon dioxide, and methanol being the actual degradation agent, as methanol consumed by the cleavage of siloxane bonds is replenished by the reaction of water with dimethyl carbonate. However, quantum chemical calculations have also suggested a direct participation of dimethyl carbonate through the formation of a complex with the simultaneous participation of the carbon atom of the carbonyl group and the oxygen atom of the methoxy group [122].

In 2015, Enthaler and colleagues, together with students from a Berlin high school, demonstrated the depolymerization of a PDMS rubber (silicone baking cup) at 200 °C with a fatty alcohol (1-decanol) in the presence of a ferric chloride (FeCl_3_) (pre)catalyst [123].

More recently, Petrus and colleagues used dimethyl carbonate and methanol to depolymerize HTV silicone rubber in the presence of fatty alcohols and KF at 150 °C or a heterobimetallic aryloxide of Mg-K with a methylsalicylato ligand as a catalyst at 220 °C [124].

### 9.5. Depolymerization by Halogen-Containing Cleavage Agents

Acid halides are another option for the depolymerization of polysiloxanes. In the early 1960s, Ashby showed that chlorine-terminated poly(dimethylsiloxane) could be depolymerized with benzoyl chloride in the presence of catalytic amounts of ferric chloride at 175 °C to give dichlorodimethylsilane and 1,3-dichlorotetramethyldisiloxane [125].

**Figure 13:**
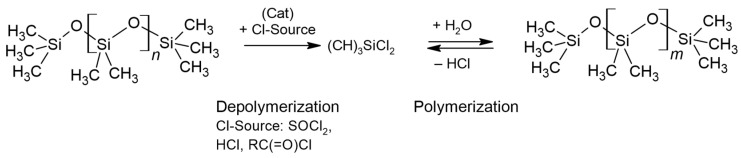
Recycling by cleavage with halogen-containing agents (schematic representation).

Recently, Enthaler showed that benzoyl chloride in combination with catalytic amounts of ferric fluoride was a suitable depolymerization agent for long-chain or branched PDMS at 190 °C [126] (see Figure 13). Alternatively, benzoyl fluoride in the presence of a zinc salt, such as 5 mol% zinc triflate, Zn(OTf)_2_, can be used to lower the decomposition temperature to 150 °C [127]. The low-boiling degradation products difluorodimethylsilane and 1,3-difluoro-1,1,3,3-tetramethyldisiloxane can be collected by distillation and repolymerized without further processing.

The above processes of activating siloxane bonds in PDMS using iron or zinc catalysts and cleaving them with benzoyl fluoride or benzyl chloride to produce silicon-containing monomers typically require high temperature, high pressure, long reaction times, and produce unwanted byproducts [128].

Another disadvantage of benzoyl fluoride as a degradation agent is its high cost. However, using ferric chloride as a (pre)catalyst, Enthaler and Kretschmer showed that not only the reaction temperature could be reduced to 100–130 °C, the catalyst loading to 2.5 mol%, and the reaction time to 1 h; but also, the benzoyl fluoride could be replaced by a mixture of benzoyl chloride and potassium fluoride, allowing for benzoyl fluoride to be formed in situ. Another alternative is to use benzoic anhydride and potassium fluoride as depolymerization agents, since acid anhydrides react with potassium fluoride to form acid fluorides and the corresponding potassium salts [129,130].

Boron halides can also be used to depolymerize polysiloxanes to fluorosilanes. Boron trifluoride diethyl etherate (BF_3_-OEt_2_) as a depolymerization agent has the advantage of not requiring a metal catalyst because it activates the Si-O bond as a Lewis acid and acts as a cleavage agent. It is also a less expensive source of fluorine than benzoyl fluoride. Using this depolymerization agent, Döhlert, Pfrommer, and Enthaler demonstrated a low-temperature depolymerization process capable of degrading linear, hydroxy-terminated PDMS oligomers at 100 °C and crosslinked PDMS at 120 °C in yields up to 85% to obtain difluorodimethylsilane and 1,3-difluoro-1,1,3,3-tetramethyldisiloxane [131,132].

However, this process requires large amounts of BF_3_-OEt_2_ catalyst (0.75–2.0 equivalents per polymer repeat unit) to achieve the desired yields. In addition, it is complicated by the formation of boron-containing byproducts that require complex isolation techniques. Finally, the depolymerization of silicones to chlorinated or fluorinated silane or siloxane is undesirable from an environmental, handling, and waste avoidance perspective, especially for chemical companies that do not have the expertise to handle these chemicals because they do not have the Müller–Rochow process capability in-house.

However, fluoride remains one of the leading candidates for depolymerizing siloxanes. Researchers with Brook [133] and Laine [128] studied the depolymerization of silicone-based materials using tetrabutylammonium fluoride (TBAF) and observed the efficient cleavage of siloxane bonds at room temperature using catalytic amounts. Laine’s team used this catalyst in tetrahydrofuran (THF) to depolymerize silicone copolymer resins and sprayed the resulting solution onto a surface. Heat treatment of the wet coating resulted in the recovery of a polymeric network.

Recently, Rupasinghe and Furgal proposed a process for decomposing polysiloxanes at low (ambient) temperatures in the presence of catalytic amounts of TBAF in organic high-swell solvents, with best results being obtained for THF [134,135]. The authors demonstrated the effectiveness of the catalyst by converting model compounds and commercially available silicone fluids and elastomers to cyclomers—primarily D_4_, D_5_, and D_6_—in as little as 30 min through an equilibration process. Other formulation components, such as calcium carbonate fillers, did not appear to hinder the depolymerization reaction. Upon completion of the depolymerization process, the catalyst was quenched by the addition of CaCl_2_ to remove active fluoride ions and prevent redistribution of the cyclic species.

Building on these findings, a group of researchers recently proposed the use of tetrabutylammonium difluorotriphenylsilicate (TBAT) as a more robust and stable catalyst for the depolymerization of polysiloxanes [136]. TBAT has the advantage of being soluble in many organic solvents and stable up to 170 °C, which allows for a wide range of fluorine-catalyzed reaction conditions. The authors demonstrated the efficiency of this catalyst in the depolymerization of a crosslinked, addition-cured silicone in THF at room temperature and in cyclohexanone at 100 °C.

In a presentation at the 10th European Silicon Days, Monteil and this team briefly mention a process for recycling a wide variety of silicones (oils, gums, resins, and even crosslinked elastomers, including silicone waste) into chlorosilane monomers using a source of chlorine and a small amount of a metallic catalyst [137]. The process is said to operate at low temperature (<60 °C) and, depending on the type of silicone processed, almost quantitative yields of Si(CH_3_)_2_Cl_2_, Si(CH_3_)_3_Cl, and/or SiR(CH_3_)Cl_2_ are obtained. Apparently, a corresponding patent has been filed [138], showing a depolymerization protocol carried out at 40 °C with various silicones using GaCl_3_ as metal salt (0.5 mol%) and BCl_3_ as metal chloride in toluene or dichloromethane solvent.

### 9.6. Depolymerization by Acid Anhydrides

**Figure 14:**
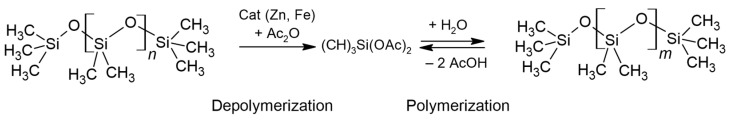
Recycling by cleavage with acid anhydrides (schematic representation).

Another feedstock recycling option for poly(dimethylsiloxanes) is to convert them to diacetoxydimethylsilanes. It has been known since the late 1950s that acid anhydrides can cleave siloxane bonds in the presence of a suitable catalyst (see Figure 14).

In 1959, Bailey and O’Connor [139] were granted a patent that claimed a process for preparing acyloxysilicon compounds by cleaving alkyl siloxanes with acetic anhydride in the presence of catalytic amounts of sulfuric acid. Interestingly, the nature of the alkyl siloxane is not restricted and could be cyclic, linear, or crosslinked. The teaching of the patent highlights the fact that the diacetoxydimethylsiloxane, after isolation, hydrolysis, and dehydration, could be repolymerized to dialkylpolysiloxanes. The example in the patent shows the deoligomerization of octamethylcyclotetrasiloxane by heating it under reflux conditions at a temperature of 136 °C to 147 °C for 40 h. The reaction results in a high yield of diacetoxydimethylsilane, acetoxydimethylsiloxyacetoxydimethylsilane, and bis(acetoxydimethylsiloxy)dimethylsilane, which together account for approximately 80% of all reaction products.

As an alternative to the use of sulfuric acid as a catalyst, depolymerization of PDMS can also be carried out in the presence of metal salts. For example, Borisov, Sviridova, and colleagues [140,141,142] describe the conversion of cyclic dimethylsiloxanes with acetic anhydride in the presence of catalytic amounts of iron (III) chloride or zinc (II) chloride to short-chain α, ω-acetoxysiloxanes in a few hours at 150 °C. However, their method yielded less than 5% of the desired diacetoxydimethylsilane.

Using ferric chloride as a catalyst, Enthaler was able to isolate diacetoxydimethylsilane after depolymerizing PDMS at 140–180 °C for 16–24 h [126]. However, significant amounts of acetic anhydride are required—two equivalents for each dimethylsiloxy unit reacted—and the yield of diacetoxydimethylsilane is modest for industrial purposes. In addition, the use of a highly corrosive iron halide makes the process further unattractive because most chemical reactors are made of high-alloy stainless steel that is not resistant to pitting and crevice corrosion.

To overcome the disadvantage of diacetoxydimethylsilane, which requires the laborious and uneconomical separation of acetic acid from the aqueous phase after hydrolysis, Enthaler and his team further developed this depolymerization concept using caproic (hexanoic) acid anhydride with a ferric chloride catalyst at 200 °C over a period of 24 h, since fatty acids are easier to separate from the aqueous phase [143]. The resulting product mixture contained hexanoate-terminated linear oligomers with mono- to tetrameric siloxane units. The authors note that this method converts silicone oils into the corresponding short-chain silicones in good yields but reaches its limits when high molecular weight, crosslinked silicones, such as those found in baking trays, are used.

Knott and colleagues at Evonik developed and patented a process that converts silicone waste into acetoxymethylsilanes and/or acetoxy-terminated siloxane by treating it at 120–130 °C with acetic anhydride and/or acetoxysiloxane and at least one Brønsted acid plus acetic acid [144]. Preferably, the Brønsted acids used are protic acids with a pK_a_ of less than −1.30. An example in the patent shows the depolymerization of shredded samples of opaque silicone tubing by heating them with decamethylcyclopentasiloxane (D_5_), acetic anhydride, acetic acid, and trifluoromethanesulfonic acid for 6 hours. After only about 45 min, the reaction mixture is an opaque, homogeneous liquid with no noticeable solids. The ^29^Si NMR spectrum confirms the reaction product as a linear α,ω-diacetoxypolydimethylsiloxane oligomer. The same chemistry can be utilized in the de-siliconization of release liners based on cellulose, polyethylene terephthalate (PET), and monoaxially or biaxially oriented polypropylene (MOPP/BOPP) carriers [145].

Another example illustrates the depolymerization of a white (acetoxy-cured) silicone sealant by heating the cut-up samples to 130 °C with D_5_, acetic anhydride, acetic acid, and concentrated sulfuric acid. After 2.5 h, more acetic anhydride is added. After an additional 6 h at 130 °C and cooling to 100 °C, potassium acetate is added to neutralize the sulfuric acid. After cooling the liquid, the white solids (not characterized by the inventors, but probably consisting of silica and titanium dioxide) are separated with a filter press. Again, the ^29^Si NMR spectrum confirms that the liquid reaction product is a linear α,ω-diacetoxypolydimethylsiloxane oligomer. After adding water and sodium hydrogen carbonate (pH ≈ 10) and heating the mixture to 80 °C for 6 h, the repolymerized silicone phase is separated from the alkaline aqueous phase and dried over sodium sulfate, and the ^29^Si NMR spectrum shows the product to be α,ω-dihydroxypolydimethylsiloxane.

Another example demonstrates the depolymerization of a red/orange silicone baking tray by heating cut samples to 120 °C with D_5_, acetic anhydride and trifluoromethanesulfonic acid while stirring. All silicone pieces are completely dissolved after about one hour. The ^29^Si NMR spectrum corresponds to a linear α,ω-diacetoxypolydimethylsiloxane oligomer.

The advantages of the Evonik process, as shown in the examples, are that the processing equipment does not need to be pressure-resistant and highly corrosion-resistant.

## 10. Current Industrial Practice of Chemical Silicone Recycling

While mechanical recycling processes for silicone elastomers are well established, their use remains limited. They generally result in downcycling, which means accepting a loss in value of the recovered material due to poorer product quality.

The potential for recycling chemical feedstocks is much greater, but it is only beginning to emerge. The vast majority of silicone elastomer waste—mainly post-industrial grades—is recycled into cyclic and linear siloxane oligomers or silicone oils; only a few companies (CHT (CHT Group offers the textile soft handle agent Tubingal^®^ Rise and Beausil^®^ RE AMO 919 EM, a microemulsion of a sugar modified siloxane (for use as ingredient in personal care products)) and Evonik (Evonik offers TEGO^®^ RC 2000 LCF (low carbon footprint), an UV LED curable release coating, made from recycled materials, for release liners in the label industry)) are currently attempting to cover a larger part of the value chain through upcycling (here, the term “upcycling” refers to the conversion of silicone waste into chemicals that have a higher market value than linear or cyclic oligomers or pyrolysis oils) [52] by offering specialized products and explicitly advertising the sale of recycled silicones.

The most widely used method for recycling silicones globally is still catalytic depolymerization into cyclomers at relatively low temperatures, from room temperature to about 180 °C, using strong Brønsted acids or bases [7,146].

In China, the cracking of silicone rubber waste using alkali hydroxide, most commonly KOH, as a catalyst was the first industrial processing method to recover cyclic siloxane oligomers. The advantages of this method are the high quality of the recovered cyclic species and comparatively low corrosion of the process equipment, but there are several drawbacks, namely, high catalyst consumption and low yield (if no co-catalyst is used), and a poor safety record, as local overheating can lead to combustion and explosion accidents [48,147].

The latter problem seems to be most prevalent when the depolymerization is performed in air at normal pressure, such as in the process described by Zhengqi [148], because then at temperatures above about 300 °C in the presence of KOH, oxidative demethylation of PDMS occurs quite rapidly [65,149], potentially leading to an explosion [150].

If the silicone waste is first soaked in an organosilicon solvent with a boiling point not higher than 80 °C, depolymerization can be performed in an alkaline medium at a temperature of 50–60 °C within 8–12 h [151]. According to Muzafarov, this method provided the basis for the development in Russia of an efficient system for recycling silicone rubber waste into a liquid polysiloxane that can be used in the manufacture of various rubber compounds [10].

Sulfuric acid acidolysis is commonly used, not just in China, to recycle silicone, often at temperatures up to 250 °C [152,153], because sulfuric acid can dissolve silicone and break the siloxane bond at the same time [147]. There are two variants of this process, one using concentrated acid and the other using diluted acid.

While sulfuric acid is inexpensive and its effectiveness as a catalyst increases with its concentration, there are serious disadvantages to its use: the acid is difficult to handle and tends to heavily corrode stainless steel reactor vessels resulting in a high equipment replacement rate, it cannot be effectively recycled and is difficult to dispose of, the yield of cyclic oligomers is low (~40–45%), and the overall composition of the cyclic oligomers is not stable, as the content of various silicone monomers varies widely [143]. In addition, concentrated sulfuric acid may also cleave the silicone–carbon bond [154].

Although the dilute sulfuric acid process has the advantages of higher cyclic oligomer recovery rate and even lower acid cost, it still suffers from severe equipment corrosion and difficulty in sulfuric acid waste disposal. In addition, the catalytic degradation process is slow—a single batch reaction typically takes more than 10 h—resulting in a low equipment utilization rate.

Depending on the specific process used, common methods for chemical recycling of silicones can generate emissions that, if not properly managed, can release a range of pollutants, including hazardous solids, highly alkaline or acidic waste streams, volatile organic compounds, and greenhouse gases.

## 11. Examples of Silicone Recycling and Upcycling

### 11.1. Silicone-Coated Fabrics

Silicone-coated fabrics based on polyamide, polyester, or fiberglass are widely used in a variety of industries—from airbags to fabric belts, from tent covers to architectural membranes—and are becoming increasingly popular as PFOA- and PFAS-free materials.

Airbags represent a significant volume of end-of-life (EoL) waste. In the United Kingdom alone, 4000 metric tons of silicone-coated fabrics were recovered from EoL airbags or airbag production waste in 2007 [155]. Recycling of this waste is generally aimed at reusing the polyamide and is done by selectively degrading or dissolving the silicone so that it separates from the fabric. To achieve this, alkali hydroxide [156]; mixtures of alkali hydroxide and a phase transfer catalyst (tetrabutylammonium chloride) [157]; mixtures of alkali hydroxide, alcohol, and surfactants [158]; as well as mixtures of Lewis acid (BF_3_) and glycol [159]; or mixtures of alkali hydroxide, tertiary amine, and surfactant [160] can be used.

In the late 2000s, researchers at the CHT Group developed a process for recycling the silicone component of airbags based on quaternary ammonium surfactants and an alkali metal hydroxide [155]. By treating the silicone-coated polyamide fabric in a pressure-resistant rotary washing machine with didecyldimethylammonium chloride, distearyldimethylammonium chloride, 1,2-propanediol, and sodium hydroxide at 135 °C for 120 min, 99.5% of the applied silicone coating could be removed from the polyamide fabric as a medium-viscosity silicone oil. According to the inventors, this recycled product has been used in the synthesis of new silicone products.

### 11.2. Silicone Sealants

Silicone waste, including cured silicone sealants and adhesives, can be upcycled using Evonik’s patented acetoxy oligomer process [161]. Linear polysiloxanes bearing acetoxy groups with an average chain length of n = 14 are obtained by digesting silicone waste for six hours at a reaction temperature of 120 °C with the addition of appropriate amounts of acetic anhydride, trifluoromethanesulfonic acid, and acetic acid. The addition of acetic anhydride is of crucial importance for the acetoxy functionalization of the siloxane.

A significant advantage of the process is that it allows for the separate recycling of polyethylene and silicone materials from a single stream of used silicone adhesive or sealant cartridges. By subjecting shredded pieces of used cartridges to thermal digestion in the acidic reaction medium, cured silicone residues in the cartridge can be completely separated from the polyethylene. The acid-catalyzed depolymerization of the silicone to a low-viscosity liquid allows for the fillers in the sealant formulation to be easily separated from the polyethylene chips. After depolymerization, the polyethylene chips are removed from the liquid siloxane by filtration through a coarse screen, and the filler can then be separated from the liquid phase by settling, for example.

The silicone oligomers may be utilized as starting material to produce two types of upcycled products: first, linear Si-O-C-linked silicone surfactants (polydimethylsiloxane-polyoxyalkylene block copolymers of the ABA structural type), for example, for subsequent use in PU foam stabilizers or as a paint additive, and second, lower-modulus sealants.

For the manufacture of polyether silicone surfactants, it is essential that the acetoxy bearing siloxane oligomers reach a thermodynamic equilibrium determined by the total cyclomer content. This can be achieved by performing the reaction initially within a temperature range of 40–120 °C and then, to ensure complete equilibration, within a temperature range of 140–160 °C, with a reaction time of 4–10 h. The corresponding polyether siloxanes are obtainable by reaction of the acetoxy siloxane oligomers with polyetherols in the presence of a base as described in a separate patent application [162].

In the manufacture of lower-modulus sealants, acetoxysiloxane oligomers are added to the silanol-terminated PDMS polymer prior to the addition of the crosslinker during the compounding process. According to the inventors, this method can be used with both acetoxy- and neutral-curing silicone sealants. The inventors attribute the lower modulus observed for these modified silicone sealants to a chain extension of the linear silanol-terminated PDMS polymer prior to crosslinking. However, based on reactivity considerations, we consider this to be an unlikely scenario. More likely is the formation of unreacted (“dead”) polymer ends in the cured network.

The “classic” acidolysis reaction with concentrated sulfuric acid, trifluoromethanesulfonic acid, methanesulfonic acid, etc., can also be used to recycle or upcycle cured silicone sealant and shredded parts of used cartridges containing silicone residues, as shown by Knott and Dudzik of Evonik [163]. With the addition of appropriate modifiers, cured silicone sealants and other silicone waste can be converted to alkoxysiloxanes, hydrogensiloxanes, chlorosiloxanes, polydimethylsiloxanes, and/or vinylsiloxanes with average chain lengths of about 20–30 by solventless, acid-catalyzed depolymerization at atmospheric pressure. For example, trimethylsilyl-terminated polydimethylsiloxane can be obtained by refluxing cured silicone sealant with decamethylcyclopentasiloxane (D_5_) and hexamethyldisiloxane (MM) in the presence of concentrated sulfuric acid at 120 °C for 4 h. Using diethoxydimethylsilane as the modifier instead yields α,ω-diethoxypolydimethylsiloxane.

## 12. Implications of Potential Regulation of Silicone Cyclomers D_4_ to D_6_ as Persistent Organic Pollutants (POPs) under the Stockholm Agreement

Thermoplastics are the primary focus of regulatory efforts due to their dominance in global plastics production. Silicone waste, while representing a small fraction of total plastic waste, comes with unique regulatory challenges. As discussed below, the silicone chemical recycling industry faces regulatory uncertainties, particularly in the European Union. Clear policy support is needed to encourage the development of chemical recycling technologies for silicones and facilitate their expansion.

In June 2018, the European Chemicals Agency (ECHA) adopted Decision ED/61/2018 to include D_4_, D_5_, and D_6_ in the Candidate List for REACH Authorization (Annex XIV), as they have been identified as substances of very high concern (SVHC) due to their persistent, bioaccumulative, and toxic (PBT), or very persistent and very bioaccumulative (vPvB) properties.

Finally, on 9 November 2023, after a legal battle in which silicone manufacturers argued to overturn the 2018 decision, the European Court of Justice confirmed the classification of these cyclomers as substances of very high concern (SVHC), upholding the previous restrictions implemented by the ECHA. This ruling is final, and no further appeal is possible. According to this ruling, the silicone cyclomers D_4_ to D_6_ shall not be placed on the market (for professional or consumer uses):

(a) As substances;
(b) As constituents of other substances (except polymers as defined in REACH Regulation (EC) No. 1907/2006 [164]) in a concentration equal to or higher than 0.1% *w*/*w*;
(c) As constituents of mixtures in a concentration equal to or greater than 0.1% *w*/*w*.

However, the Court’s decision also includes several exemptions. In the context of silicone production and recycling, the most important of these is that the restriction on placing these siloxane cyclomers on the market does not apply to their industrial use as a monomer in the production of silicone polymer [165].

The EU Regulation 2019/1021 (“POP Regulation”) [166] sets out detailed requirements for the Member States of the European Union regarding the production, placing on the market, use, and release of persistent organic pollutants (POPs). The regulation implements the Stockholm Convention on Persistent Organic Pollutants, to which the EU is a Party, and the United Nations Economic Commission for Europe (UNECE) Protocol on POPs. Regulation 2019/1021 stipulates that POPs can only be recovered from waste for the sole purpose of destruction. The recovery of the cyclic compounds for use as monomer in the production of silicone polymers appears to be acceptable under certain conditions, as this use is specifically exempted by the European Court ruling.

However, the European Commission now intends to submit a proposal for a nomination of the same cyclic siloxanes as POPs to Annex B of the United Nations Stockholm Convention. Consultation of stakeholders on draft reports for these substances have been closed on 10 August 2023. The Stockholm Convention aims to protect human health and the environment from persistent organic pollutants. Since the goal of the Stockholm Convention is the global elimination of these substances, such a classification could not only affect the availability of new silicone products but could also result in restrictions or bans that would affect the ability to ship, recycle, and safely dispose of the waste [167].

While the Stockholm Convention generally exempts POPs present as “unintentional trace contaminants” (UTCs) in products from the requirements of Annexes A and B, there is no precise definition of UTCs. If the allowable UTCs in waste were set at an unworkable level, all waste containing silicone polymers would be classified as hazardous waste and would have to be incinerated.

Listing D_4_, D_5_, and D_6_ under the Stockholm Convention, if not exempted as UTCs, would be detrimental to recycling and circularity. Recycling processes that generate cyclomers D_4_ to D_6_ could then be prohibited, which would contradict the EU’s circular economy objective of increasing recycling rates.

There are also implications for trade flows of waste containing cyclic siloxanes. Transboundary movements of hazardous wastes are subject to control under the Basel Convention [168] and must be minimized. However, the recycling of silicones will only be technically and economically feasible on a sufficiently large scale, which will require the transportation of waste streams globally and throughout Europe.

To ensure a viable future for silicone recycling (and related industry investment) in the EU, it is critical that polysiloxanes containing UTCs are green-listed under Annex III of the new EU Regulation on Shipments of Waste [169]. The green-listing of UTC-containing polysiloxanes will streamline the process of transporting silicone waste across EU borders for recycling. This will increase the availability of recyclable silicone material and encourage more companies to enter the recycling market. Simplified cross-border shipping and reduced costs can create new market opportunities, allowing for a wider range of companies to benefit from the silicone recycling industry. Finally, increasing recycling rates will reduce the amount of silicone waste sent to landfills, thereby minimizing environmental impact and conserving natural resources for future generations.

## 13. Market Situation: Silicone Recycling at an Inflection Point?

While the global market for silicones in 2024 is estimated at about 3 million metric tons [12,170] with a sales value of USD 22 billion [171], the immediately addressable global recycling market is much smaller, perhaps 100,000–150,000 metric tons, as the feedstock is predominantly elastomers, mostly from post-industrial and some post-consumer waste (In market reports, the mix is generally estimated to be two-thirds post-industrial and one-third post-consumer).

In comparison, we estimate that 35,000 to 40,000 metric tons of silicone waste will be chemically recycled worldwide this year, with Asia accounting for 60–70%, North America for 20–30%, and Europe for 10–15%. Assuming an average efficiency (yield) of 50% in the recycling processes and a selling price of USD 2/kg for dimethylsiloxane cyclomers (DMC), this corresponds to a sales volume of USD 35–40 million. These estimates are based on the geographic location of the recycler, not the origin of the silicone waste. Large quantities of silicone waste (approximately 6000–10,000 tons) are shipped from Europe to Southeast Asia because current legislation does not prohibit such exports.

Table 3 shows the leading players in the global silicone chemical recycling market that we are aware of. We estimate that there are currently one to two dozen players worldwide, with new players entering the market all the time. The number of active silicone recycling companies in China is probably much higher than we know.

The players are generally small and medium-sized companies with few employees; however, these medium-sized companies can play a dominant role in the market regionally (example: ECO, USA, with a recycling volume of 8000–10,000 metric tons per year [172]). Processes used commercially by smaller companies are generally not patented by them and are likely to be based on patents that have expired.

According to a recent study by the German Chemical Industry Association [173], the demand for recycled silicone raw materials far exceeds the current supply. Despite this shortage, customers are unwilling to pay above market prices for recycled materials. One of the “lessons learned,” according to the study, is that recycled products are slow to gain market acceptance because potential buyers are concerned about their availability and quality; however, it is believed that this can be overcome if the products are associated with a recognized brand.

Collaboration and co-development of innovation capabilities between start-up companies focused on silicone recycling and established silicone raw material producers are currently on the rise, a trend that could lead to better integration of recycling into the value chains of large corporations in the future.

The two main drivers for the incumbent silicone manufacturers to pursue value chain partnerships are

1. Investment de-risking: Corporate investment decisions often favor known risks (optimization of existing processes or business units) over unknown risks (new capabilities and processes); by partnering with SMEs, disruptive business models can be developed and tested with low financial risk.
2. Increased profitability and flexibility: The inability of large corporations to internally develop cost-effective services and low-volume start-up operations (waste collection, sorting, and recycling) due to high labor and overhead costs and organizational inflexibility makes partnering with SMEs that offer low-cost structure and attractive organizational flexibility.

Examples of such collaborations include the recently announced partnership between Dow Silicones and Circusil [174], and the collaboration between Elkem (a subsidiary of Sinochem Holdings) and external partners in the REPOS (Ressourcement Polymères Silicones) project, which was awarded the CEFIC Circularity Commendation 2023 by the European Chemical Industry Council in 2023 [175]. The consortium led by Elkem includes the Laboratory of Catalysis, Polymerization, Processes, and Materials (CP2M) and the Institute of Polymer Materials Engineering (IMP) of the Universities of Lyon [97,137,138] as well as two SMEs, Activation (https://www.activation.fr/ (accessed on 30 May 2024)) and Processium (https://www.processium.com/en/ (accessed on 30 May 2024)).

In recent years, several digital platforms have emerged as business-to-business, cross-sector marketplaces that provide collaborative value networks for the circular economy. For example, the German startup Cirplus (https://www.cirplus.com/ (accessed on 30 May 2024)), founded in 2018, brings together more than 3000 plastics processors, product manufacturers, recyclers, and waste management companies from over 100 countries in Europe’s largest digital procurement platform for recycled plastics. Several silicone manufacturers already offer virtual platforms for trading virgin polymers and finished products; therefore, it is likely that they will integrate trading of recycled materials into their digital business models in the future.

At present, post-consumer silicone recycling is limited to the collection—by the original manufacturer or specialized service companies—of items such as baking pans, table mats, pacifiers and teething rings, tubes, ear plugs, wristbands, oven mitts, and similar extruded or molded parts, resulting in relatively low collection volumes.

Access to sorted post-consumer plastic waste is one of the biggest barriers to scaling up recycling in general. Using artificial intelligence, a robotic sorting system based on the deep learning software GAIN has been in use since 2018 at a sorting facility for lightweight packaging in Leipzig, Germany, to remove silicone cartridges from the packaging waste stream, a task that commercially available near-infrared separators, for example, are unable to perform [176].

These efforts to remove sealant cartridges are aimed at keeping the plastic packaging waste stream silicone-free, as silicone has a negative impact on the recycling and processing of plastics. As a result, each year, millions of consumer and professional sealant cartridges end up in landfills [177] or must be incinerated.

In the future, the use of external cavity quantum cascade lasers (EC-QCLs) may significantly improve the identification of silicone waste in post-consumer and demolition waste. Quantum cascade lasers (QCLs) provide a polarized, coherent, and high-power light source in the mid-infrared (MIR) region of the electromagnetic spectrum. Combined with an external cavity (EC) grating for tuning, EC-QCLs provide broadband spectral tuning over hundreds of wavenumbers, making them versatile tools for remote and real-time chemical sensing using MIR backscattering spectroscopy [178].

Silicone recycling initiatives are gaining traction in the market. In 2022, Henkel launched a global innovation call for technologies to separate plastic from silicone waste in used silicone cartridges [179] to enable separate recycling of polyethylene packaging and silicone.

Effective recycling of silicone sealants is important because they represent the largest share of global silicone sales, with building and construction being the largest segment [12,170]. Cured sealants removed from the substrate during resealing operations are currently disposed of as commercial or site waste. By identifying and segregating silicone sealants, they could be effectively recycled.

Currently, when insulating glass is recycled [180], the metal spacers are removed and recycled, but the primary and secondary sealants are either left to burn off in the float glass furnace [181] or disposed of as organic waste along with laminating films, labels, and larger plastic particles [182]. However, concepts are being developed to dissemble returned insulating glass units so that each component can be re-used, re-manufactured or recycled, and valorized, including silicone [183].

## 14. Key Challenges of Scaling Up the Recycling of Silicone Chemicals

The transition from small-scale to industrial-scale chemical recycling of silicones poses several key challenges, including the need to overcome technical, economic, and logistical barriers, as outlined below:

Feedstock supply: Ensuring a secure and consistent supply of feedstock requires a well-organized system for collecting, transporting, and storing silicone waste to maintain a steady flow of material.

Feedstock variability: While small-scale operations often handle more homogeneous waste streams, at larger scales, significant variability in feedstock quality may be encountered, which can include variability in composition, contamination levels, and physical states such as liquids, solids, cured, and uncured.

Process equipment design: Scaling up silicone waste processing requires designing process equipment that can efficiently handle large volumes while maintaining optimal reaction, separation, and purification conditions.

Economic viability: Identifying and implementing cost-saving measures is essential for maintaining economic viability, which in turn ensures the scalability of the recycling process.

By-product utilization: Effective management and utilization of byproducts and waste streams generated during the recycling process are essential for sustaining the recycling operation, as it enables the creation of value from previously discarded materials. A particular challenge here is the high filler content of silicone sealants and elastomers.

Quality assurance: Implementing real-time monitoring and control systems that can incorporate advanced sensors, automation, and data analytics is vital for ensuring consistent product quality and optimal process efficiency at scale.

Regulatory compliance: At larger scale, meeting regulatory requirements for emissions, waste management, and safety standards becomes increasingly complex and demanding, requiring rigorous compliance measures to avoid potential penalties.

Product distribution: Establishing effective distribution channels for recycled silicone products is essential to ensure timely and cost-effective delivery to customers, thereby maximizing market reach and competitiveness.

Overcoming these technical and commercial hurdles requires a collaborative effort that combines expertise from multiple disciplines, including chemical engineering, materials science, industrial automation, and marketing.

## 15. Outlook

How will the silicone recycling market evolve? Initially, recyclers will continue to focus on post-industrial elastomer waste streams, as these are the easiest to separate into discrete materials. In addition, material streams that produce the least amount of low-value byproducts during recycling will be favored. For example, silica-filled platinum-catalyzed addition-cured silicones (LSRs) are likely to be preferred over calcium carbonate-filled silicone sealants, even though they are available in smaller quantities, because the lower filler content makes recycling easier. In addition, although the platinum catalyst is a tiny component of the formulation (concentration in the ppm range), it represents a high value, making the recovery of the recycling byproduct more attractive [184].

The future success of silicone recycling will depend not only on the cost structure of the recycling process and the availability of sufficient quantities of silicone waste from post-consumer and post-industrial collections, but also on comprehensive end-of-life recycling that returns as many raw materials as possible to production cycles in the long term.

To create a stable market for secondary raw materials, it will be necessary to put primary and secondary raw materials on an equal regulatory footing. However, this will require—at least for many applications—that all raw materials, regardless of their origin (primary or secondary), be subject to the same quality requirements. Regulatory recognition of chemical recycling is therefore of particular importance, not only for the recovery of mechanically non-recyclable materials in general [185], but also regarding the purity of the secondary raw materials obtained.

At the regulatory level, it will also be necessary to revise current specifications and update standards that still block the use of secondary raw materials [186] (this effort has been initiated with the development of DIN SPEC 91446 [187] in Germany; for further necessary steps, see [188]).

Much of the value creation potential is expected to come from service-based business models and the transformation of value networks. Digitalization will play a crucial role in sorting (post-industrial and post-consumer waste), reverse logistics (just-in-time collection of waste from customers), and the marketing of quality-tested and certified secondary materials (recyclates) via digital trading platforms (marketplace).

It is therefore likely that truly disruptive changes in the value chain will only come from new cooperative business models in which different companies bundle different products and services and offer them jointly to customers.

To operate cost-effectively, recyclers depend on a steady supply of silicone waste, so contracts with companies that supply such waste are paramount. As a result, the business models of the larger recyclers already include services designed to increase customer loyalty. In North America, for example, some companies offer a collection service for their customers’ silicone waste. In addition, partnerships between silicone producers and recyclers, between recyclers and waste suppliers, and between recyclers and off-takers are emerging and will become increasingly important for disruptive business models for large-scale industrial operations in the future.

Companies will continue to set ambitious targets that prioritize recycling and waste reduction, in line with evolving regulatory requirements. Tightening import regulations and evolving environmental and chemical safety standards will drive a shift toward localized (area-specific) recycling initiatives. While these changes present challenges, they also offer opportunities to improve the sustainability and efficiency of silicone recycling processes.

In the construction and building segment, information on recycled or upcycled silicone products and their environmental impact will be integrated into online platforms with building product information relevant to sustainability and building certification as a basis for decision-making [189,190]. Green building certifications programs, such as LEED (Leadership in Energy and Environmental Design) and BREEAM (Building Research Establishment Environmental Assessment Method), will likely require documentation of recycled content and environmental impact. Eco-labels that indicate the percentage of recycled content and the environmental impact of silicone products will become the standard. Recycled silicone products will have environmental product declarations (EPDs) that highlight their reduced environmental footprint compared to virgin materials. Government agencies will be updating building codes to incorporate sustainability requirements, mandating the use of recycled materials, including silicone, to ensure compliance. The management of information on recycled or upcycled silicone products and their environmental impact in the construction and building segment will involve a multi-faceted approach, comprising certification and labeling schemes, digital platforms, regulatory compliance, market education, and awareness campaigns.

As a result, the market for silicone recycling will continue to grow at an above-average rate, driven by the global push for recycling-oriented practices, the resulting customer demand, and tighter government regulations.

Silicone recycling may not provide an immediate return on investment for global integrated silicone manufacturers, but collaboration with innovative start-up companies will help them gain a competitive advantage and strengthen their market position in the long term.

Realistically, however, unless significant advances are made in the emerging fields of microbial [147,191] or enzymatic [192,193] recycling, especially of low-concentration silicones, a substantial portion of the waste silicone stream from today’s applications will not be recycled for decades to come. This is due to the high barriers to recovery of silicones present at low levels (e.g., as additives in paints or personal care products) in wastewater, municipal solid waste, or construction debris.

## 16. Conclusions

Adopting a circular economy approach is paramount in promoting the sustainability of silicone elastomers in various industries, including building and construction. At a qualitative level, it is now well understood that the recycling of silicone products contributes to the reduction in greenhouse gas emissions and energy consumption associated with silicone production, the reduction in landfill waste, and the conservation of natural resources. As recycling techniques continue to be implemented on an industrial scale, these environmental benefits will become increasingly quantifiable.

Physical (mechanical) recycling results in some loss of structural or aesthetic properties each time a silicone product is recycled and is therefore considered “downcycling”. Chemical recycling technologies allow for closed-loop processes that promise endless virgin-grade recycling. The oligomers obtained by depolymerizing end-of-life silicones can be repolymerized by seamlessly integrating them back into the basic silicone manufacturing process. Chemical recycling also enables product upcycling—the conversion of silicone waste into higher-value products.

Most of the current research in the chemical recycling of silicones is focused on the development of new catalytic systems to improve the efficiency of polysiloxane depolymerization, but not on the development of recycling processes themselves. It is obvious that more effort is needed to develop an industrially viable and attractive route for recycling both liquid and solid silicones on a large scale.

While the amount of chemically recycled silicone is still small—estimated at about 40,000 tons in 2024—and recycling is carried out by a cottage industry of small and medium-sized companies with few employees, collaboration and co-development of innovation capabilities between start-ups and established silicone raw material producers are on the rise.

We expect these value chain partnerships and coalitions to lead to the construction of state-of-the-art, commercial-scale silicone recycling plants in the 2024–2026 timeframe. Digital platforms will continue to emerge as business-to-business, cross-sector marketplaces that provide collaborative value networks for the circular economy and are likely to be integrated into the business models of silicone feedstock producers. The integration of Internet of Things (IoT) devices and artificial intelligence (AI) will automate the identification and sorting of silicone waste, increasing the efficiency and accuracy of recycling operations. Digital platforms will enable companies to find recycling or upcycling partners for low-volume waste streams that would otherwise need to be scrapped. Furthermore, from a transaction platform perspective, as more companies join these exchanges, planning uncertainty will decrease, material availability will increase, and product quality will become more stable. The next step is likely to be the exchange of know-how among market participants to create a proprietary knowledge space within a platform.

The regulatory environment continues to be dynamic. Recent international import restrictions have accelerated the need to improve domestic markets for recyclable materials. The listing of D_4_, D_5_, and D_6_ under the Stockholm Convention, if not exempted as Unintentional Trace Contaminants (UTCs), would be detrimental to recycling and circularity. Recycling processes that generate cyclomers D_4_ to D_6_ could then be prohibited, which would be contrary to the EU’s circular economy objective of increasing recycling rates. Furthermore, to ensure a viable future for silicone recycling on a larger, industrial scale (and the associated industry investment) in the European Union, it is critical that polysiloxanes containing UTCs be green-listed under Annex III of the new EU Waste Shipment Regulation.

Since the global building and construction (B&C) industry is one of the largest users of silicone products, but has a poor recycling record, it is essential to develop strategies to promote the recyclability of silicone products that go beyond the development of in-house closed-loop chemical recycling processes.

Architects, engineers, manufacturers, and other stakeholders must join efforts to design and manage buildings and building components with replacement and deconstruction in mind (“Design for Deconstruction and Materials Reuse”). Silicone products need to be designed with recyclability in mind. There is a growing demand for removable sealants and adhesives that facilitate both repair and recycling at the end of a product’s life (“Debonding on Demand”). Educating end users by providing clear instructions on how to dispose of recyclable silicone products can encourage responsible behavior and increase recycling rates. Silicone gaskets, sealants, and adhesives are too often disposed of in mixed construction and demolition waste. Millions of cartridges are used worldwide each year. Measures need to be developed to reuse or recycle empty or partially empty cartridges.

## Figures and Tables

**Table 1:** Silicone properties, product forms, and examples of applications in the building and construction industry. Note: The characteristics required for successful performance depend on the specific application. The key features shown here are the absolute minimum required for all applications listed for a product form.

Silicone Features	Product Forms and Key Features	Applications (Examples)
Durability (outstanding longevity of life) Weather resistance Flexibility Extreme operating temperature range Chemical resistance Water repellence and resistance Low water absorption Thermally stability Low surface tension Ease of application Fire resistance Electric insulation Non-yellowing Mostly low toxicity	Sealants (1–3)	Glazing, expansion joints, connection joints
	Adhesives (1–3)	Structural glazing, structural bonding, insulating glass edge seal
	Coatings (2, 6)	Façade weatherproofing, roof coating
	Foams (3, 8)	Fire penetration seals, insulation of district-heating pipes
	Films (1, 3, 13)	Soundproof laminated glass interlayer, structural bonding
	Gels (8, 12, 13)	Light guidance systems (“light pipelines”)
	Fluids (2, 6, 9)	Water-repellant, sealer
	Additives (7, 8, 9)	PU foam stabilizer, impact modifier, surfactant

**Table 2:** Overview of methods in commercial use or under development.

Depolymerization Method	Auxiliary Chemical	Intermediates	Typical Catalyst	Conversion of Intermediates Prior to Polymerization	Used on Industrial Scale
Protonic acids	Solvent	D_3_–D_6_, linear oligomers	H_2_SO_4_	No	Yes
Strong inorganic bases	Solvent	D_3_–D_6_, linear oligomers	KOH	No	Yes
Acid anhydrides	Acetic acidanhydride, Solvent	α,ω-diacetoxydimethylsiloxanes, diacetoxy dimethyl silane	Trifluoromethanesulfonic acid	Yes	Under development
Halogen containing cleavage agents	BCl_3_ Solvents	Dichlorodimethylsilane	GaCl_3_	Yes	Under development
Alcoholysis	Methanol, Solvents	Dimethyldimethoxysilane or α,ω-dimethoxydimethylsiloxanes	Strong acids or bases	Yes	Yes

**Table 3:** Leading players in the global silicone chemical recycling market. Note: players marked with (*) are global traders/recyclers who collect waste at their location and have it recycled in another geographic area, typically in Asia. All URLs were accessed on 30 May 2024.

Area	Company	Webpage
**U.S.A.**	ECO	https://www.ecousarecycling.com/
	Circusil	https://circularsilicones.com/
	Harmony Industries	http://www.harmonyrecycling.com/
**Germany**	CHT Group	https://cht-silicones.com/
	KomRec-Recond (*)	https://komrec-recond.de/de/home
	Wandaa (*)	https://silicone.wandaa.com/
	GW United Chemicals (*)	https://www.gwunitedchem.com/silcone-recyling
**India**	Supreme Silicones	http://supremesilicones.com/
	Ecovalley Silicones	https://www.ecovalleyindia.com/
**Thailand**	Recycle Engineering	https://www.recycleengineering.com/en/
**Singapore**	Global Serve	http://www.theglobalserve.com/
**China**	Yangzhou Hongyuan Chemical Industry New Material Company	https://www.yzhyxc.com/
	Jiangshan Huashun Organic Silicone Company	http://m.zjhuashun.com/
	Shenzhen Shengtian Silicon Rubber Material Technology	http://shengtian88.com/en/

## References

[1] Weinhold F., West R. (2011). The nature of the silicon–oxygen bond. Organometallics.

[2] Moraru I.-T., Petrar P.M., Nemeş G. (2017). Bridging a knowledge gap from siloxanes to germoxanes and stannoxanes. A theoretical natural bond orbital study. J. Phys. Chem. A.

[3] Dankert F., von Hänisch C. (2021). Siloxane coordination revisited: Si−O bond character, reactivity and magnificent molecular shapes. Eur. J. Inorg. Chem..

[4] Mordor Intelligence, Silicone Market Size & Share Analysis—Growth Trends & Forecasts (2024–2029). https://www.mordorintelligence.com/industry-reports/silicone-market.

[5] EMR CLAIGHT Expert Market Research, Global Silicones Market Size, Share, Trends, Growth—Report: 2024–2032. https://www.expertmarketresearch.com/reports/silicone-market-report.

[6] Merget R., Bauer T., Küpper H.U., Philippou S., Bauer H.D., Breitstadt R., Bruening T. (2002). Health hazards due to the inhalation of amorphous silica. Arch. Toxicol..

[7] Rupasinghe B., Evingür G.A., Pekcan Ö. (2022). Recycling silicone-based materials: An overview of methods. Application and Characterization of Rubber Materials.

[8] Rupasinghe B. (2022). Transformations of Siloxane-Based Materials toward a Reuse and Recycling Loop: Catalytic Methods and Photochemistry. Ph.D. Thesis.

[9] Rupasinghe B., Furgal J.C. (2021). Degradation of silicone-based materials as a driving force for recyclability. Polym. Int..

[10] Muzafarov A.M., Bystrova A.V., Vasilenko N.G., Ignat’eva G.M. (2013). New approaches in silicon production and recycling for sustainable future. Russ. Chem. Rev..

[11] Elmanovich I.V., Sizov V.E., Zefirov V.V., Kalinina A.A., Gallyamov M.O., Papkov V.S., Muzafarov A.M. (2022). Chemical recycling of high-molecular-weight organosilicon compounds in supercritical fluids. Polymers.

[12] Global Silicones Council (GSC), Construction. https://globalsilicones.org/explore-silicones/benefits-uses/construction/.

[13] Klosowski J.M., Wolf A.T. (2015). Sealants in Construction.

[14] De Buyl F. (2001). Silicone Sealants and Structural Adhesives. Int. J. Adhes. Adhes..

[15] De Buyl F., Hayez V., Harkness B., Kimberlain J., Shephard N., Dillard D.A. (2023). Advances in structural silicone adhesives. Advances in Structural Adhesive Bonding.

[16] Hopewell J., Dvorak R., Kosior E. (2009). Plastics recycling: Challenges and opportunities. Philos. Trans. R. Soc. Lond. B Biol. Sci..

[17] Santos G., Esmizadeh E., Riahinezhad M. (2024). Recycling Construction, Renovation, and Demolition Plastic Waste: Review of the Status Quo, Challenges and Opportunities. J. Polym. Environ..

[18] Gui L. (2020). Recycling infrastructure development under extended producer responsibility in developing economies. Prod. Oper. Manag..

[19] Andriot M., Chao S.H., Colas A., Cray S., de Buyl F., DeGroot J.V., Dupont A., Easton T., Garaud J.L., Gerlach E., Roger D.J., Mario G. (2007). Silicones in Industrial Applications. Inorganic Polymers.

[20] Dvornic P.R., Lenz R.W. (1990). High Temperature Siloxane Elastomers.

[21] European Commission Communication on The European Green Deal. https://commission.europa.eu/document/daef3e5c-a456-4fbb-a067-8f1cbe8d9c78_en.

[22] World Economic Forum The World Needs a Circular Economy. https://www.weforum.org/agenda/2020/01/the-world-needs-a-circular-economy-lets-make-it-happen/.

[23] Deloitte, BDI Zirkuläre Wirtschaft. Herausforderungen und Chancen für den Industriestandort Deutschland. https://www2.deloitte.com/content/dam/Deloitte/de/Documents/risk/Zirkul%C3%A4re%20Wirtschaft%20Studie_Deloitte%20und%20BDI.pdf.

[24] European Commission A New Circular Economy Action Plan for a Cleaner and More Competitive Europe, Brussels (2020). https://eur-lex.europa.eu/legal-content/EN/TXT/?uri=COM:2020:98:FIN.

[25] Chandra G., Maxim L.D., Sawano T., Chandra G. (1997). The silicone industry and its environmental impact. Organosilicon Materials. The Handbook of Environmental Chemistry.

[26] Hayez V. (2024). Personal communication.

[27] Cutri E., Hayez V., Vetterli J., Willareth P. Silicones—An important enabler of sustainable design. Proceedings of the Glass Performance Days.

[28] Deutsche Rohstoffagentur (DERA) in der Bundesanstalt für Geowissenschaften und Rohstoffe (BGR), Silizium. https://www.deutsche-rohstoffagentur.de/DERA/DE/Aktuelles/rohstoff_silizium.html.

[29] Bauer S. (2003). RW SILICIUM—Geschichte, Stand, Ausblick. https://www.deutsche-rohstoffagentur.de/DERA/DE/Downloads/Si-vorkshop-Geschichte.pdf;jsessionid=C8F67E8B421195CDD8414ABD8A583E4A.internet962?__blob=publicationFile&v=2.

[30] Critical Raw Materials Alliance, Silicon Metal. https://www.crmalliance.eu/silicon-metal.

[31] (2024). Innovative Pilot for Silicon Production with Low Environmental Impact Using Secondary Aluminum and Silicon Raw Materials, CORDIS. https://cordis.europa.eu/project/id/869268.

[32] Mahmoodinia M., Farooq H., Røe T., Svenum I.-H., Venvik H.J. (2023). Effect of copper catalyst content and zinc promoter on carbon formation in the direct synthesis of methylchlorosilanes. Ind. Eng. Chem. Res..

[33] Zhang P., Zhang D., Dong J., Chen G., Li J. (2022). Direct synthesis of methylchlorosilanes: Catalysts, mechanisms, reaction conditions, and reactor designs. Org. Process Res. Dev..

[34] Seyferth D. (2001). Dimethyldichlorosilane and the direct synthesis of methylchlorosilanes. The key to the silicones industry. Organometallics.

[35] Hurd C.B. (1946). Studies on siloxanes: I. The specific volume and viscosity in relation to temperature and constitution. J. Am. Chem. Soc..

[36] Stark F.O., Falender J.R., Wright A.P., Wilkinson G., Stone F.G.A., Abel E.W. (1982). Silicones. Comprehensive Organometallic Chemistry.

[37] Schliebs R., Ackermann J. (1987). Chemie und Technologie der Silicone I. Chem. Unserer Zeit.

[38] Roberts J.M., Belowich M.E., Peterson T.H., Bellinger E., Syverud K., Laitar D.S., Sidle T. (2020). Homoconjugated acids as low cyclosiloxane-producing silanol polycondensation catalysts. ACS Omega.

[39] Cypryk M., Matyjaszewski K., Möller M. (2012). Polymerization of cyclic siloxanes, silanes, and related monomers. Polymer Science: A Comprehensive Reference.

[40] Rösch L., Weidner R., Buschow K.H.J., Cahn R.W., Flemings M.C., Ilschner B., Kramer E.J., Mahajan S., Veyssière P. (2001). Polymerization chemistry of silicones. Encyclopedia of Materials: Science and Technology.

[41] Kendrick T.C., Parbhoo B.M., White J.W., Allen G., Bevington J.C. (1989). Polymerization of cyclosiloxanes. Comprehensive Polymer Science and Supplements.

[42] Currie J., Griffith P., Herron W., Taylor R. (2000). Process for Producing a Silicone Polymer. U.S. Patent.

[43] Gilson J.-M., de la Croi Habimana J. (1993). Method of Making Organopoysiloxanes. U.S. Patent.

[44] Colas A., Curtis J., Modjarrad K., Ebnesajjad S. (2013). Silicones. Handbook of Polymer Applications in Medicine and Medical Devices.

[45] Boonstra B.B., Cochrane H., Dánnenberg E.M. (1975). Reinforcement of silicone rubber by particulate silica. Rubber Chem. Technol..

[46] Cochrane H., Lin C.S. (1993). The influence of fumed silica properties on the processing, curing, and reinforcement properties of silicone rubber. Rubber Chem. Technol..

[47] Aranguren M.I., Mora E., Macosko C.W., Saam J. (1994). Rheological and mechanical properties of filled rubber: Silica-silicone. Rubber Chem. Technol..

[48] Xu X.-M., Tao X.-L., Zheng Q. (2008). Influence of surface-modification for calcium carbonate on the interaction between the fillers and polydimethylsiloxane. Chin. J. Polym. Sci..

[49] Ghosh A., Rajeev R.S., Bhattacharya A.K., Bhowmick A.K., De S.K. (2003). Recycling of silicone rubber waste: Effect of ground silicone rubber vulcanizate powder on the properties of silicone rubber. Polym. Eng. Sci..

[50] Scott D.W. (1946). Equilibria between linear and cyclic polymers in methylpolysiloxanes. J. Am. Chem. Soc..

[51] Semlyen J.A. (1976). Ring-chain equilibria and the conformations of polymer chains. Mechanisms of Polyreactions-Polymer Characterization. Advances in Polymer Science.

[52] Vollmer I., Jenks M.J.F., Roelands M.C.P., White R.J., van Harmelen T., de Wild P., van der Laan G.P., Meirer F., Keurentjes J.T.F., Weckhuysen B.M. (2020). Beyond mechanical recycling: Giving new life to plastic waste. Angew. Chem. Int. Ed..

[53] Cited in: Knott W., Dudzik H., Schäfer D. (2022). Process for Recycling Silicones. U.S. Patent.

[54] ISO 14067:2018 Greenhouse gases—Carbon footprint of products—Requirements and guidelines for quantification, International Standardization Organization, Geneva, Switzerland.

[55] Cheng A. Life Cycle Assessment of Silicone Oil & Carbon Savings. Proceedings of the Silicone Expo USA.

[56] Plastics Technology Dow, Circusil Collaborating on Silicone Recycling Facility. https://www.ptonline.com/news/dow-circusil-collaborating-on-silicone-recycling-facility.

[57] Environmental management—Life cycle assessment—Principles and framework.

[58] Environmental management—Life cycle assessment—Requirements and guidelines.

[59] Lewis R.N. (1948). Methylphenylpolysiloxanes. J. Am. Chem. Soc..

[60] Scala L., Hickam W. (1958). Thermal and oxidative degradation of silicones. Ind. Eng. Chem..

[61] de Buyl F., Yoshida S., van Driel W.D., Yazdan Mehr M. (2022). Degradation Mechanisms of Silicones. Reliability of Organic Compounds in Microelectronics and Optoelectronics.

[62] Friebe R., Weber W., Sockel K.-H. (1999). Activator for the Depolymerization of Polysiloxanes Which Are Crosslinked, Optionally Contain Fillers and/or Are Uncrosslinked. U.S. Patent.

[63] Haas H., Snyder M. (2004). Thickened Silicone Dissolving Agent. U.S. Patent.

[64] Camino C., Lomakin S.M., Lazzari M. (2001). Polydimethylsiloxane thermal degradation—Part 1. Kinetic aspects. Polymer.

[65] Dvornic P.R., Jones R.G., Ando W., Chojnowski J. (2000). Thermal Properties of Polysiloxanes. Silicon-Containing Polymers.

[66] Aleksandrova Y.A., Nikitina J.S., Pravednikov A.N. (1968). Study of the mechanism of polydimethylsiloxane thermal decomposition. Vysokomol. Soedin..

[67] Rode V.V., Verkhotin V.A., Rafikov S.R. (1969). Study of the thermal degradation and stabilization of polydimethylsiloxane. Vysokomol. Soyed.

[68] Thomas T.H., Kendrick T.C. (1969). Thermal analysis of polydimethylsiloxanes. I. Thermal degradation in controlled atmospheres. J. Polym. Sci. Part A-2.

[69] Grassie N., Macfarlane I.G. (1978). The thermal degradation of polysiloxanes—I. Poly(dimethylsiloxane). Eur. Polym. J..

[70] Patnode W., Wilcock D.F. (1946). Methylpolysiloxanes. J. Am. Chem. Soc..

[71] Jovanovic J.D., Govedarica M.N., Dvornic P.R., Popovic I.G. (1998). The thermogravimetric analysis of some polysiloxanes. Polym. Degrad. Stab..

[72] Allan D., Radzinski S.C., Tapsak M.A., Liggat J.J. (2016). The thermal degradation behavior of a series of siloxane co-polymers—A study by thermal volatilization analysis. Silicon.

[73] Lewicki J.P., Mayer B.P., Alviso C.T., Maxwell R.S. (2012). Thermal degradation behavior and product speciation in model poly(dimethylsiloxane) networks. J. Inorg. Organomet. Polym..

[74] Camino G., Lomakin S.M., Lageard M. (2002). Thermal polydimethylsiloxane degradation. Part 2. The degradation mechanisms. Polymer.

[75] Narisawa M. (2010). Silicone resin applications for ceramic precursors and composites. Materials.

[76] He C., Li B., Ren Y., Lu W., Zeng Y., He W., Feng A. (2018). How the crosslinking agent influences the thermal stability of RTV phenyl silicone rubber. Materials.

[77] Papkov V.S., Bulkin A.F., Zhadanov A.A., Slonimskii G.L., Andrianov K.A. (1977). Investigation of the thermal oxidation of polydimethylsiloxanes. Polym. Sci. USSR.

[78] Ruaigaj A., Krajnc M., Šebenik U. (2017). Kinetic study of thermal degradation of polydimethylsiloxane: The effect of molecular weight on thermal stability in inert atmosphere. Polym. Sci..

[79] Cypryk M., Apeloig Y. (2002). Mechanism of the acid-catalyzed Si−O bond cleavage in siloxanes and Siloxanols. A theoretical study. Organometallics.

[80] Humphrey B.J., Wasserman H.H. (1954). Liquefaction of Silicone Rubber, Gums and Products Thereof. U.S. Patent.

[81] (1956). N.N. (Midland Silicones). A Process for Reclaiming Siloxane Elastomers. British Patent.

[82] Burkhardt J., Louis E. (1981). Process for Preparing Cyclic Dimethylpolysiloxanes. U.S. Patent.

[83] Greenlee T.W. (1992). Process for Recycling Silicone Scrap and Products Relating Thereto. U.S. Patent.

[84] Xu H., Wu W., Zhang F., Qi H., Feng Y. (2012). Recycling Method of Waste Silicone Rubber. Chinese Patent.

[85] Miller C.J., Ryan W.P. (1973). Method of Making Cyclopolysiloxanes Containing Silanic Hydrogen. U.S. Patent.

[86] Crivello J.W., Lee J.L. (1990). Method for Making Cyclic Poly(siloxane)s. U.S. Patent.

[87] Knott W., Dudzik H. (2022). Process for Producing Endcapped, Liquid Siloxanes from Silicone Wastes. U.S. Patent.

[88] Hyde J.F. (1948). Method of Preparing Silicones and Product Thereof. U.S. Patent.

[89] Hunter M.J., Hyde J.F., Warrick E.L., Fletcher H.J. (1946). Organo-silicon polymers. The cyclic dimethyl siloxanes. J. Am. Chem. Soc..

[90] Andrianov K.A., Shapatin A.S., Zhigalin G.Y., Golyaeva I.M., Kleinovskaya M.A., Safronova O.A., Trufanov A.G., Ufimtsev N.G., Kopylov V.M. (1980). Method for Producing Organocyclosiloxane. Soviet. Union Patent.

[91] Shapatin A.S., Simanenko E.A., Zhigalin G.Y., Trufanov A.G., Golyaeva I.M. (1982). Process for Producing Organocyclosiloxanes. Soviet. Union Patent.

[92] Grinblat M.L., Ivanova L.S., Kissin K.V., Plaksina Z.G., Romanikhin V.B. (1998). Method of Treating Organosilicon Waste. Russian Patent.

[93] Voyloshnikov V.M., Taramasova D.R., Ezhov M.V., Shmelev I.G., Voyloshnikov A.V. (2016). Method for Obtaining Cyclosiloxanes and Low-Molecular Polydimethylsiloxane. Russian Patent.

[94] Allandrieu C., Cardinaud D. (1997). Process for the Manufacture of Cyclosiloxanes by Depolymerization of Polysiloxanes. U.S. Patent.

[95] Razzano J.S. (1995). Siloxane Cracking Process. U.S. Patent.

[96] Yang J.-K., Han J.-R., Shin J.-B., Hong J.-J. (2003). Novel Process for Decomposing Siloxane Bond-Containing Compound. European Patent.

[97] Vu D.-N., Boulegue-Mondière A., Durand N., Raynaud J., Monteil V. (2023). Back to Cyclic Monomers: Chemical Recycling of Silicone Waste Using a [Polydentate Ligand Potassium Silanolate] Complex. Green Chem..

[98] Boulegue-Mondière A., Durand N., Vu D.-N., Raynaud J., Monteil V. (2023). Organopolysiloxane Depolymerization. PCT. International Patent.

[99] Weitkamp R.F., Neumann B., Stammler H.-G., Hoge B. (2020). Synthesis and reactivity of the first isolated hydrogen-bridged silanol–silanolate anions. Angew. Chem. Int. Ed..

[100] Weitkamp R.F., Neumann B., Stammler H.-G., Hoge B. (2021). The influence of weakly coordinating cations on the O−H⋅⋅⋅O− hydrogen bond of silanol-silanolate anions. Chem. Eur. J..

[101] Weitkamp R.F. (2021). Phosphazenbasen und die Darstellung nicht-koordinierter Anionen. Ph.D. Thesis.

[102] Torgunrud J.L., Reverón Pérez A.M., Spitzberg E.B., Miller S.A. (2023). Entropy-driven depolymerization of poly(dimethylsiloxane). Macromolecules.

[103] Hsiao Y.-C., Hill L.W., Pappas S.P. (1975). Reversible amine solubilization of cured siloxane polymers. J. Appl. Polym. Sci..

[104] Doyle C.D. (1951). Silicone Resin Solutions. U.S. Patent.

[105] Schimmel K.H. (1987). Polysiloxane. 3. Aminolytische Spaltung von Polysiloxan-Modellverbindungen. Acta Polym..

[106] Schimmel K.H., Schulz J. (1987). Polysiloxane. 4. Zum Verhalten linearer Poly(dimethylsiloxane) in Diethylamine. Acta Polym..

[107] Schimmel K.H., Schröder E., Schulz J., Souvimonh T. (1988). Polysiloxane. 5. Netzwerkspaltung durch Amine. Acta Polym..

[108] Chang C.-L., Lee H.S., Chen C.K. (1999). Aminolysis of cured siloxane polymers. Polym Degrad Stab..

[109] Chang C.-L., Don T.-M., Lee H.S.-J., Sha Y.-O. (2004). Studies on the aminolysis of RTV silicone rubber and modifications of degradation products. Polym. Degrad. Stab..

[110] Chang C.-L., Lee H.S.-J., Chen C.K. (2005). Nucleophilic cleavage of crosslinked polysiloxanes to cyclic siloxane monomers: Mild catalysis by a designed polar solvent system. J. Polym. Res..

[111] Oku A., Huang W., Ikeda Y. (2002). Monomer recycling for vulcanized silicone rubbers in the form of cyclosiloxane monomers. Role of acid buffers. Polymer.

[112] Huang W., Ikeda Y., Oku A. (2002). Recovery of monomers and fillers from high-temperature-vulcanized silicone rubbers—Combined effects of solvent, base and fillers. Polymer.

[113] Ikeda Y., Huang W., Oku A. (2003). Recycling of monomers and fillers from high-temperature-vulcanized silicone rubber using tetramethylammonium hydroxide. Green Chem..

[114] Hara H., Ikeda H., Ko I., Oku A., Sawada K., Shudo K., Ikeda Y., Suto K. (2008). Method for Depolymerizing Filler-Containing Silicone Compounds. Japanese Patent.

[115] Hara H., Shudo K., Sawada K., Oku A., Ikeda H., Ko I. (2007). Method for Depolymerizing Filled Silicone Compound. Japanese Patent.

[116] Hron P., Heidingsfeldová M., Schätz M. (1980). Reclamation of Siloxanes. Czech Patent.

[117] Voronkov M.G., Shabarova Z.I. (1959). Alkoxysilanes. XIV. Cleavage of organosiloxanes by alcohols as a method of synthesis of organoalkoxysilanes. Zh. Obsh. Khim..

[118] Hirose T., Tsuji R., Ouchi K. (2001). Method for Decomposing Polysiloxane. European Patent.

[119] Okamoto M., Miyazaki K., Kado A., Suzuki S., Suzuki E. (2003). Deoligomerization of cyclooligosiloxanes with dimethyl carbonate over solid-base catalysts. Catal. Lett..

[120] Okamoto M., Suzuki S., Suzuki E. (2004). Polysiloxane depolymerization with dimethyl carbonate using alkali metal halide catalysts. Appl. Catal. Gen..

[121] Kawamoto T. (2004). Process for Recycling Silicone Compounds. European Patent.

[122] Demianenko E.M., Grebenyuk A.G., Lobanov V.V., Protsak I.S., Kozakevych R.B., Bolbukh Y.M., Tertykh V.A. (2014). Quantum chemical study on interaction of dimethyl carbonate with polydimethylsiloxane. Chem. Phys. Technol. Surf..

[123] Weidauer M., Heyder B., Woelki D., Tschiersch M., Köhler-Krützfeldt A., Enthaler S. (2015). Iron-catalyzed depolymerizations of end-of-life silicones with fatty alcohols. Resour. Effic. Technol..

[124] Petrus R., Utko J., Gniłka R., Fleszar M.G., Lis T., Sobota P. (2021). Solvothermal alcoholysis method for recycling high-consistency silicone rubber waste. Macromolecules.

[125] Ashby B.A. (1965). Process for Preparing Halogenated Organo-Silicon Materials. British Patent.

[126] Enthaler S. (2015). Iron-catalyzed depolymerization of polysiloxanes to produce dichlorodimethylsilane, diacetoxydimethylsilane, or dimethoxydimethylsilane. J. Appl. Polym. Sci..

[127] Enthaler S. (2014). Zinc-catalyzed depolymerization of end-of-life polysiloxanes. Angew. Chem. Int. Ed..

[128] Krug D.J., Asuncion M.Z., Laine R.M. (2019). Facile approach to recycling highly crosslinked thermoset silicone resins under ambient conditions. ACS Omega.

[129] Enthaler S., Kretschmer R. (2014). Low-temperature depolymerization of polysiloxanes with iron catalysis. ChemSusChem.

[130] Döhlert P., Weidauer M., Peifer R., Kohl S., Enthaler S. (2015). Introducing students to feedstock recycling of end-of-life silicones via a low-temperature, iron-catalyzed depolymerization process. J. Chem. Educ..

[131] Döhlert P., Pfrommer J., Enthaler S. (2015). Recycling concept for end-of-life silicones: Boron trifluoride diethyl etherate as depolymerization reagent to produce difluorodimethylsilane as useful commodity. ACS Sustain. Chem. Eng..

[132] Döhlert P. (2016). Depolymerisation von Polysiloxanen und die Eisenvermittelte Aktivierung Kleiner Moleküle. Ph.D. Thesis.

[133] Brook M.A., Zhao S., Liu L., Chen Y. (2012). Surface etching of silicone elastomers by depolymerization. Can. J. Chem..

[134] Rupasinghe B., Furgal J.C. (2021). Full circle recycling of polysiloxanes via room-temperature fluoride-catalyzed depolymerization to repolymerizable cyclics. ACS Appl. Polym. Mater..

[135] Furgal J.C., Rupasinghe B. (2022). Fluoride Catalyzed Polysiloxane Depolymerization. PCT Patent.

[136] Warner M.J., Kopatz J.W., Schafer D.P., Kustas J., Sawyer P.S., Grillet A.M., Jones B.H., Ghosh K. (2024). A robust depolymerization route for polysiloxanes. Chem. Commun..

[137] Vu N.D., Munsch J., Boulegue-Mondiere A., Durand N., Vovelle L., Raynaud J., Monteil V. Chemical Recycling of Silicones. Proceedings of the 10th European Silicon Days.

[138] Boulegue-Mondiere A., Durand N., Vu D.-N., Raynaud J., Monteil V. (2024). Dépolymérisation de Silicone en Organochlorosilane. French Patent.

[139] Bailey D., O’Connor T.M. (1959). Process for Producing Acyloxysilicon Compounds. U.S. Patent.

[140] Borisov S.N., Sviridova N.G. (1968). Acetoxysiloxane oligomers I. The interaction of acetic anhydride with cyclic dimethylsiloxanes. J. Organomet. Chem..

[141] Borisov S.N., Sviridova N.G., Voronkov M.G. (1968). Cleavage of cyclodimethylsiloxanes by acetic anhydride. Zh. Obshch. Khim..

[142] Voronkov M.G., Sviridova N.G., Yuzhelevskii Y.A., Borisov S.N. (1969). Splitting of cyclic methyl(3,3,3-trifluoropropyl)-, methylvinyl-, methylphenyl-, and diethylsiloxanes by acetic anhydride. Zh. Obshch. Khim..

[143] Weidauer M., Heyder B., Woelki D., Tschiersch M., Köhler-Krützfeldt A., Enthaler S. (2015). Iron-catalyzed depolymerizations of silicones with hexanoic anhydride provide a potential recycling method for end-of-life polymers: Depolymerization of silicones with hexanoic anhydride. J. Lipid Sci. Technol..

[144] Knott W., Dudzik H., Schäfer D. (2021). Method for Recycling of Silicones. European Patent.

[145] Knott W., Dudzik H., Schäfer D. (2024). Recycling of Siliconized Flat/Planar Sheets. European Patent.

[146] Kantor S.W., Grubb W., Osthoff R.C. (1954). The mechanism of the acid- and base-catalyzed equilibration of siloxanes. J. Am. Chem. Soc..

[147] Yang S., Liu Y., Zhou D. (2022). Monomer recovery and nano-silica separation from biodegraded waste silicone rubber shed of composite insulator. Front. Mater.-Sec. Polym. Compos. Mater..

[148] Zhengqi C. (1999). Organosilicon Cyclic Body Prepared by Silastic Atmospheric Cracking. Chinese Patent.

[149] Grassie N., Francey K.F., Macfarlane I.G. (1980). The thermal degradation of polysiloxanes—Part 4: Poly(dimethyl/diphenyl siloxane). Polym. Degrad. Stab..

[150] Mukherjee S.R., Paul A.K. (2001). Process for the Recovery of Methyl Polysiloxanes in the Form of Cyclosiloxanes. U.S. Patent.

[151] Vojloshnikov V.M., Shmelev I.G., Karimova D.R. (2011). Method of Treating Organosilicon Waste. Russian Patent.

[152] Chen J. (2014). Process for Preparing Dimethylcyclosiloxane Mixture from Internal Decomposition of Organic Silicone Waste by Acid Method. Chinese Patent.

[153] Yu D., Mai B. (2024). A Production Method and Device for DMC. Chinese Patent.

[154] Sommer L.H., Barie W.P., Gould J.R. (1953). Kinetics and mechanism of methyl—Silicon cleavage by sulfuric acid. J. Am. Chem. Soc..

[155] Nickel F., Hilton D.M. (2011). Removing the Silicone Coating from Coated Fabrics and Airbags. European Patent.

[156] Ohira I. (2007). Method of Removing Silicone from Airbag Scrap Textile. Japanese Patent.

[157] Mignani G. (2010). Treatment Method for an Article Comprising a Plastic Material Covered by a Silicone Material. European Patent.

[158] Andrews R. (2008). Treatment of Polyamides. PCT Patent.

[159] Yang J.H., Lee D.J. (2012). Recycling Method of Wasted Air Bag Fabrics. Korean Patent.

[160] Mihashi T. (2017). Recycling Method of Air Bag Scrap Cloth Made of Nylon. Japanese Patent.

[161] Knott W., Dudzik H., Schäfer D. (2022). Upcycling Method for Processing Silicone Waste. European Patent.

[162] Knott W., Dudzik H., Favresse P. (2021). Linear Acetoxy Group Bearing Siloxanes and Secondary Products. European Patent.

[163] Knott W., Dudzik H. (2022). Method for the Production of Terminated, Liquid Siloxanes from Silicone Waste. European Patent.

[164] European Commission, Regulation (EC) No 1907/2006 of the European Parliament and of the Council of 18 December 2006 (Amended Version). https://eur-lex.europa.eu/eli/reg/2006/1907/2023-08-06.

[165] Gazerro C. D4, D5 and D6: Substances of Very High Concern. https://toxhub-consulting.com/2023/12/14/d4-d5-and-d6-substances-of-very-high-concern/.

[166] European Commission, Regulation (EU) 2019/1021 of the European Parliament and of the Council of 20 June 2019 on Persistent Organic Pollutants (recast) (Text with EEA Relevance). https://eur-lex.europa.eu/eli/reg/2019/1021/oj.

[167] Evonik Industries AG, Questions & Answers for Cyclic Siloxanes D4, D5 and D6. https://products.evonik.com/assets/68/08/FAQ_Questions_and_Answers_SVHC_listing_D4_D5_D6_EN_196808.pdf.

[168] UNEP, Basel Convention on the Control of Transboundary Movements of Hazardous Wastes and Their Disposal. https://www.basel.int/Portals/4/Basel%20Convention/docs/text/BaselConventionText-e.pdf.

[169] Council of the EU, Press Release: Waste shipments: Council and Parliament Reach Agreement on More Efficient and Updated Rules (17 November 2023 03:40). https://www.consilium.europa.eu/en/press/press-releases/2023/11/17/waste-shipments-council-and-parliament-reach-agreement-on-more-efficient-and-updated-rules/#:~:text=The%20regulation%20covers%20the%20export,EU%20and%20non%2DOECD%20countries.

[170] Cheng A. (2021). An overview of the silicone recycling process. Rubber World.

[171] Grand View Research, Silicone Sealants Market Size, Share & Growth Report, 2023–2030. https://www.grandviewresearch.com.

[172] ECO USA Silicone Recycling. https://www.ecousarecycling.com/about-us/.

[173] Verband der Chemischen Industrie e. V Chemie³ Guide: Launching the Circular Economy in the Chemical Industry (May 2022). https://www.chemiehoch3.de/fileadmin/user_upload/Chemie3_Guide_to_Circular_Economy.pdf.

[174] Dow, Circusil Embark on Silicone Rubber Recycling Journey. https://www.rubbernews.com/silicone/dow-circusil-open-silicone-recycling-plant-kentucky.

[175] European Chemical Industry Council (CEFIC), REPOS—Innovative Depolymerization Technology For Silicones. https://cefic.org/responsible-care/2023-european-responsible-care-awards/repos-innovative-depolymerization-technology-for-silicones.

[176] Artificial Intelligence in Waste Management. https://www.recovery-worldwide.com/en/artikel/artificial-intelligence-in-waste-management-3506287.html.

[177] Gort I., Haffmans S. Kitkokers in een Circulaire Economy (Sealant Tubes in a Circular Economy), Partners for Innovation (2017). https://kidv.nl/media/rapportages/kitkokers_in_een_circulaire_economy.pdf?1.1.2-rc.1.

[178] Ostendorf R., Butschek L., Hugger S., Fuchs F., Yang Q., Jarvis J., Schilling C., Rattunde M., Merten A., Grahmann J. (2016). Recent advances and applications of external cavity-QCLs towards hyperspectral imaging for standoff detection and real-time spectroscopic sensing of chemicals. Photonics.

[179] Anonymous (2022). Striving for sustainability—Recycling silicone sealant cartridges. Adhes. Sealants Ind..

[180] Rose A., Nothacker K., Gassman A. Recycling of Flat Glass in the Building Industry—Analysis of the Current Situation and Derivation of Recommendations for Action, ift-Research Report, ift Rosenheim, 11/2019. https://www.irbnet.de/daten/kbf/kbf_e_F_3202.pdf.

[181] Devlin K. (2022). Flat glass recycling. Glass Mag..

[182] Mills J. (2024). Reiling commissions glass recycling plant in Germany. Glass Int..

[183] Babic E., Dodd G., Resnick M., Entwistl D., Gibson A., Hayez V., Erlbacher E. Reversing the supply chain: Recovery of IGU components. Proceedings of the GPD Glass Performance Days.

[184] Feix T., Fadhil A.A., Troegel D. (2024). Leaching of catalyst platinum from cured silicone elastomers: A preliminary study for comparing reagents. Hydrometallurgy.

[185] Carbonminds, Agora Industrie Klimapositive Chemie. Leitbild für Klimaschutz, Effizienz und Wirtschaftlichkeit. https://www.agora-industrie.de/fileadmin/Projekte/2022/2022-02_IND_Climate_Positive_Chemistry_DE/2022-11-04_Praesentation_Webinar_Climate_positive_chemistry.pdf.

[186] Jenet A., Lamperti Tornaghi M., Tsionis G., Sejersen A., Moseley P., De La Fuente Nuno A., Wrobel M., Hobbs G., Guldager Jensen K., Chevauche C. (2024). Circular Technologies in Construction. https://data.europa.eu/doi/10.2760/876431.

[187] DIN SPEC 91446:2021-12. Classification of Recycled Plastics by Data Quality Levels for Use and (Digital) Trading. https://www.beuth.de/de/technische-regel/din-spec-91446/346496956.

[188] Standardization Roadmap: Circular Economy. https://www.din.de/resource/blob/906910/0d691bed63405ae85f281336ed71162c/standardization-roadmap-circular-economy-data.pdf.

[189] Chen Z., Chen L., Zhou X., Huang L., Sandanayake M., Yap P.-S. (2024). Recent technological advancements in BIM and LCA integration for sustainable construction: A review. Sustainability.

[190] Cavalliere C., Dell’Osso G.R., Pierucci A., Iannone F. (2018). Life cycle assessment data structure for Building Information Modelling. J. Clean. Prod..

[191] Pascual C., Lebrero R., Cantera S. (2023). Toward a sustainable and cost-efficient biological-based platform for siloxanes removal. Crit. Rev. Environ. Sci. Tec..

[192] Sarai N.S., Fulton T.J., O’Meara R.L., Johnston K.E., Brinkmann-Chen S., Maar R.R., Tecklenburg R.E., Roberts J.M., Reddel J.C.T., Katsoulis D.E. (2024). Directed evolution of enzymatic silicon-carbon bond cleavage in siloxanes. Science.

[193] Müller W., Schröder H.C., Krasko A. (2005). Degradation and Modification of Silicates and Silicones by Silicase and Use of the Reversible Enzyme. German Patent.

